# Supplementary material for: Regulation of neuroendocrine plasticity by the RNA-binding protein ZFP36L1
Source: Nat Commun. 2022 Aug 25;13:4998. doi: 10.1038/s41467-022-31998-7 (PMC9411550; doi:10.1038/s41467-022-31998-7)
Supplement: Supplementary file 1 — Supplementary Information [file 41467_2022_31998_MOESM1_ESM.pdf]

Supplementary Information

Fig. S1

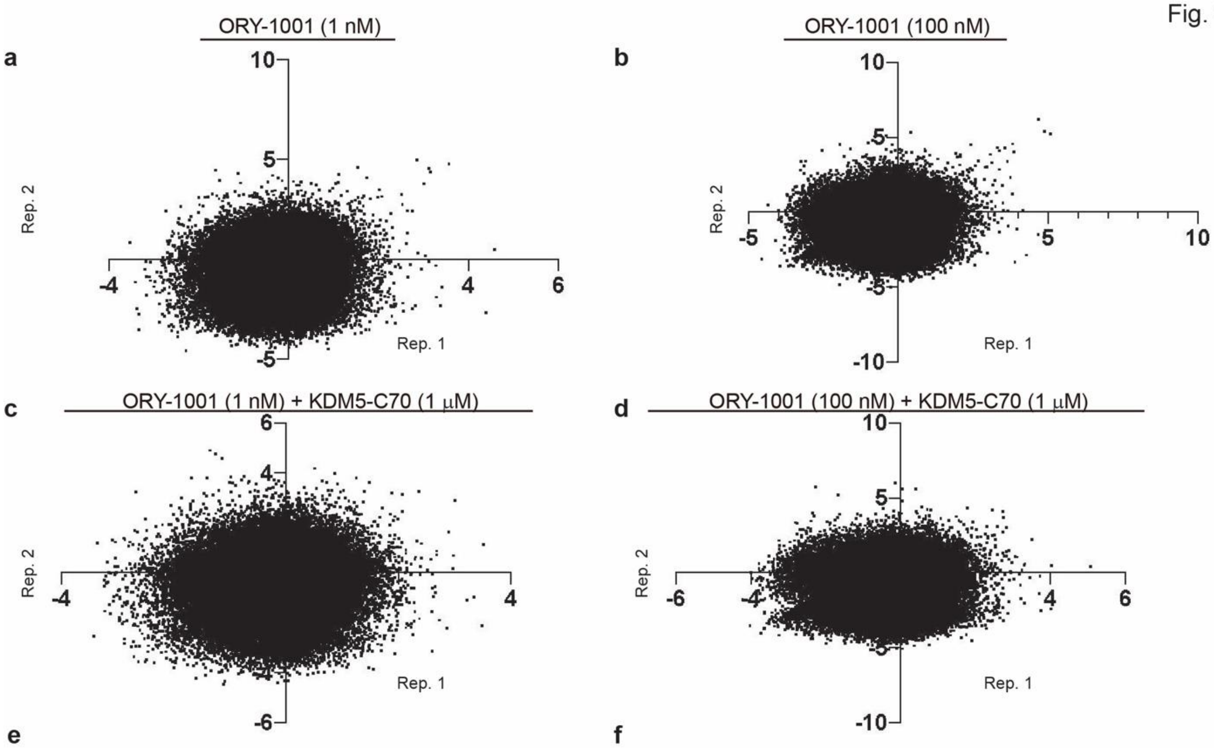

e

ORY-1001 1 nM

Gene Symbol	Ranks of perturbations	FDR	q-value
MYH9	3;5;7;8	0	0
ROCK2	1;2;4;17958	0	0
LZTR1	18;39;72;127	0	0
PAWR	12;27;45;245	0	0
**ZFP36L1**	33;44;79;12039	0	0
IRAK1	13;88;728;1092	0.00017777	0.00017777
MED12	29;53;219;22272	0.00060951	0.00060951
CSNK1A1	6;23;14465;75312	0.00386659	0.00386659
KMT2C	28;32;4539;19604	0.00722949	0.0066132
TP73	268;369;509;9193	0.0066132	0.0066132
HMGB1	43;366;1385;2755	0.00989071	0.00989071
EP300	891;1292;1517;3155	0.01608857	0.01608857
DPH5	56;65;704;10599	0.01632788	0.01632788
DAPK3	24;70;794;6921	0.02034246	0.02034246
LUZP1	193;438;833;5220	0.02154624	0.02133291

f

ORY-1001 100 nM

Gene Symbol	Ranks of perturbations	FDR	q-value
MYH9	3;5;6;8	0	0
PAWR	13;19;24;28	0	0
ROCK2	1;2;4;50486	0	0
LZTR1	7;18;30;540	0	0
**ZFP36L1**	40;277;321;592	0	0
CDYL	199;291;583;656	0	0
EP300	26;115;126;965	0.00015159	0.00013264
MEN1	76;240;529;967	0.00013264	0.00013264
IRAK1	156;417;655;1267	0.00023581	0.00023581
MYL6	75;160;313;3208	0.00233452	0.00229915
HMGB1	54;1350;1882;1968	0.00241169	0.00229915
DPH5	11;21;387;3517	0.00229915	0.00229915
MED12	10;39;421;4970	0.00261205	0.00261205
MED19	25;32;891;67435	0.00462356	0.00431532
DAPK3	22;44;502;6389	0.00431532	0.00431532

g

ORY-1001 1 nM + KDM5-C70 1 μM

Gene Symbol	Ranks of perturbations	FDR	q-value
**ZFP36L1**	3;11;53;80	0	0
ROCK2	4;9;75;25091	0	0
MYH9	262;279;331;603	0	0
DLX5	103;327;575;706	0	0
CBFB	15;71;113;30177	0.00021455	0.00021455
RHOA	83;349;391;1286	0.00035759	0.00035759
LUZP1	859;1121;1236;1673	0.00245202	0.00245202
RUNX1	169;593;1198;2857	0.01622546	0.01622546
TP73	786;1904;2893;3088	0.01978641	0.01978641
REST	49;192;712;67837	0.02188425	0.02188425
PAWR	631;721;949;3472	0.02477094	0.02477094
TSC22D2	256;1023;3314;3767	0.03057359	0.03057359
UBE2E1	157;1010;1138;62810	0.06213741	0.06213741
DAPK3	332;2009;3114;4684	0.06520833	0.06520833
P2RY10	122;131;3259;49265	0.07731005	0.07731005

h

ORY-1001 100 nM + KDM5-C70 1 μM

Gene Symbo	Ranks of perturbations	FDR	q-value
ROCK2	1;8;14;11612	0	0
**ZFP36L1**	12;22;29;267	0	0
MYH9	3;26;34;1022	0	0
CBFB	13;49;92;18226	0	0
PAWR	86;158;260;6625	0.00172252	0.00172252
CXorf49	24;48;428;624;1769;6962;52586;68869	0.00338353	0.00296059
CXorf49B	24;48;428;624;1769;6962;52586;68869	0.00338353	0.00296059
RHOA	25;255;323;17870	0.00296059	0.00296059
LUZP1	114;188;450;4113	0.00490441	0.00490441
HMGB1	169;374;1755;2380	0.00527523	0.00518714
NRBP1	17;31;5736;20619	0.00518714	0.00518714
SCFD2	99;537;600;49418	0.01067606	0.01067606
DENR	45;55;5051;27737	0.01689398	0.01689398
WWTR1	9;702;2311;3414	0.01837871	0.01830181
PGGT1B	4;64;1153;11664	0.01830181	0.01830181
DMRTC1B	16;37;1222;2165;4154;7623;39471;47793	0.02488793	0.02488793
DMRTC1	16;37;1222;2165;4154;7623;39471;47793	0.02488793	0.02488793
ZNF519	191;601;827;26960;54759	0.04300328	0.04300328
PKN2	44;123;1832;4671	0.04759605	0.04759605
YAP1	7;154;17728;45374	0.0794514	0.0794514
SUDS3	440;610;1492;29193	0.09053502	0.08964952
HMGB1P5	374;1755;2380	0.08964952	0.08964952
CASP9	30;179;15245;19272	0.0947388	0.0947388
KIAA1217	776;3220;5794;6059	0.10559429	0.10253322
REST	240;794;1650;29826	0.10253322	0.10253322

Supplementary Fig. 1. CRISPR/Cas9 Positive Selection Screen Identifies Genes Required for LSD1 Inhibitor Sensitivity in Small Cell Lung Cancer. (**a-d**) Log-fold change (LFC) of sgRNAs on day 35 relative to the early timepoint (day 11) of screen replicate 1 compared to screen replicate 2 of the screens treated with ORY-1001 (1 nM) (**a**), ORY-1001 (100 nM) (**b**), ORY-1001 (1 nM) + KDM5-C70 (1000 nM) (**c**), or ORY-1001 (100 nM) + KDM5-C70 (1000 nM) (**d**). n=2 biological replicates. (**e-h**) STARS analysis from the positive-selection CRISPR/Cas9 screen on day 35 relative to the early timepoint prior to drug treatment (day 11) of NCI-H1876 Cas9 cells infected with the Brunello sgRNA library and then treated with ORY-1001 (1 nM) (**e**), ORY-1001 (100 nM) (**f**), ORY-1001 (1 nM) + KDM5-C70 (1000 nM) (**g**), or ORY-1001 (100 nM) + KDM5-C70 (1000 nM) (**h**). For all panels, n=2 biological independent experiments. Statistical significance was calculated using a negative binominal distribution (STARS analysis) corrected for multiple hypothesis testing to yield FDRs and q-values as indicated.

Fig. S2

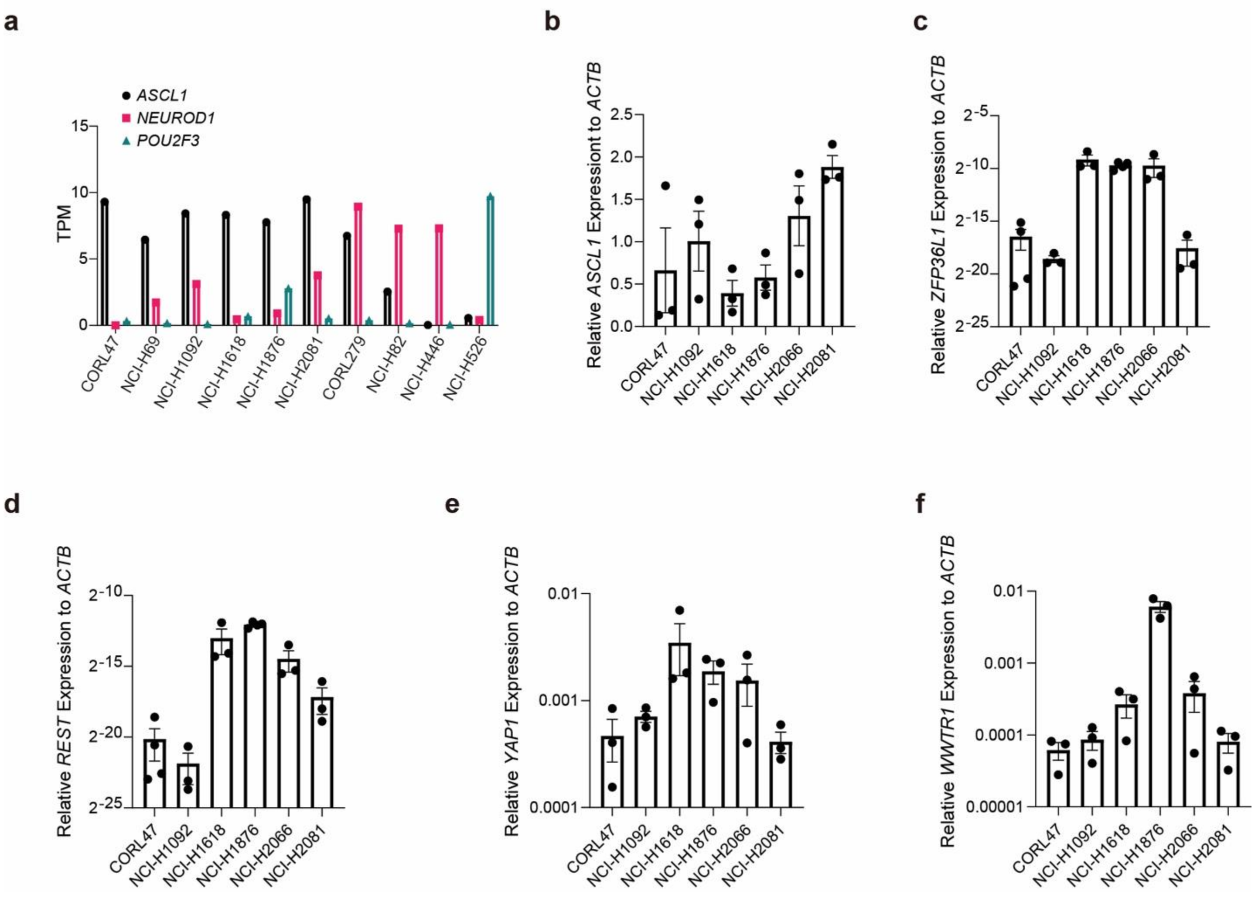

Supplementary Fig. 2. Molecular Subtype Expression and ΔC_T Values for Baseline Gene Expression in Small Cell Lung Cancer Cell Lines Used in this Study.

(a) Gene expression TPM (transcripts per million) values of *ASCL1*, *NEUROD1*, and *POU2F3* from the Cancer Cell Line Encyclopedia (CCLE). For a, mRNA expression in TPM for individual cell lines are shown. (**b-f**) ΔC_T method followed by $2^{-\Delta CT}$ to determine baseline gene expression of ASCL1 **(b),** *ZFP36L1* **(c),** *REST* **(d),** *YAP1* **(e),** and *WWTR1* **(f)** relative to *ACTB* for the small cell lung cancer cell lines indicated**.** For b,e,f n=3 biological independent experiments. For c,d, n=3 biological independent experiments for all cell lines except CORL47 and NCI-H1876 where n=4 biological independent experiments. For b-f, data are presented as mean values +/- SEM.

Fig. S3

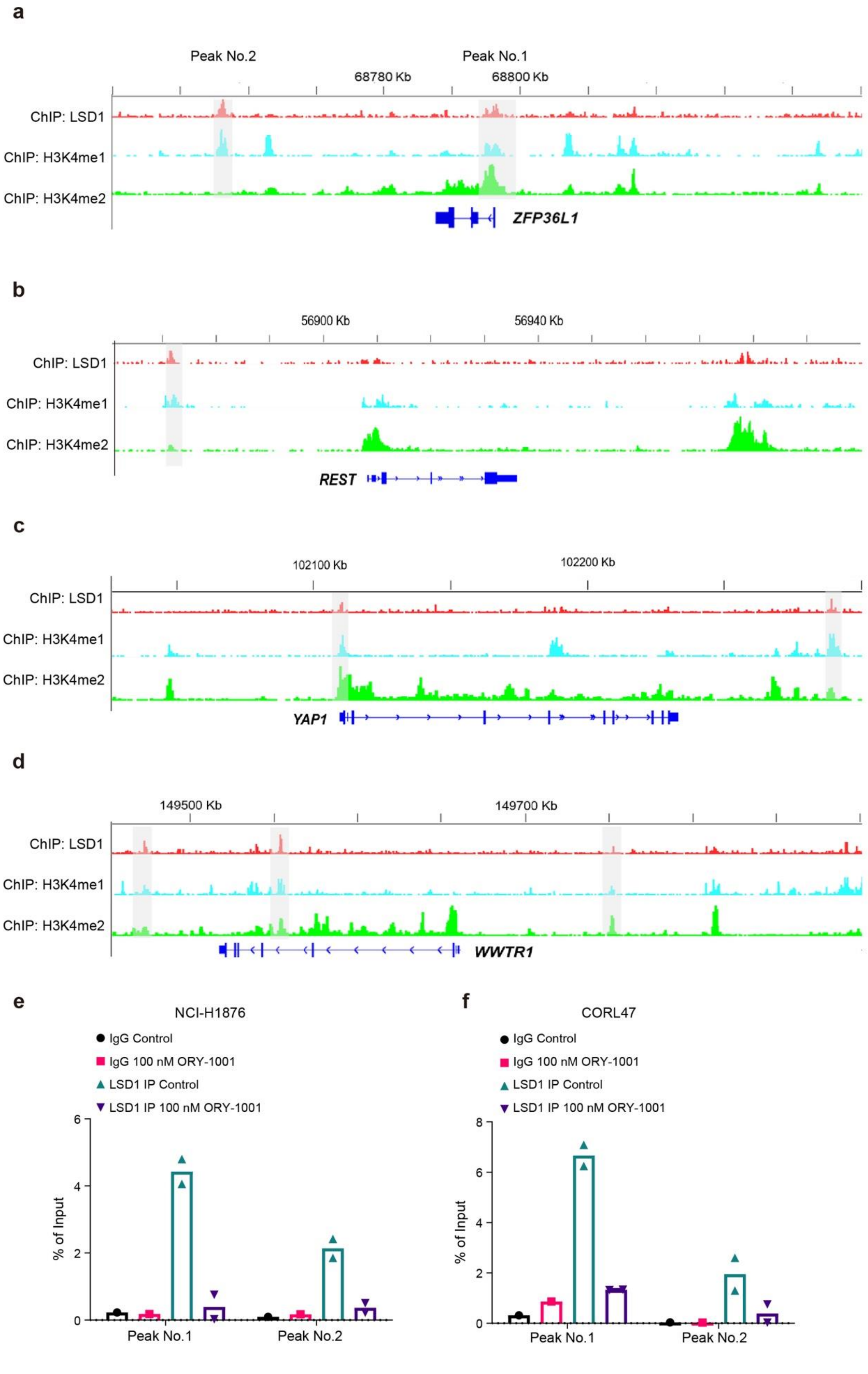

a
Peak No.2
Peak No.1
68780 Kb
68800 Kb
ChIP: LSD1
ChIP: H3K4me1
ChIP: H3K4me2
ZFP36L1
b
56900 Kb
56940 Kb
ChIP: LSD1
ChIP: H3K4me1
ChIP: H3K4me2
REST
c
102100 Kb
102200 Kb
ChIP: LSD1
ChIP: H3K4me1
ChIP: H3K4me2
YAP1
d
149500 Kb
149700 Kb
ChIP: LSD1
ChIP: H3K4me1
ChIP: H3K4me2
WWTR1
e
NCI-H1876
IgG Control
IgG 100 nM ORY-1001
LSD1 IP Control
LSD1 IP 100 nM ORY-1001
% of Input
0
2
4
6
Peak No.1
Peak No.2
f
CORL47
IgG Control
IgG 100 nM ORY-1001
LSD1 IP Control
LSD1 IP 100 nM ORY-1001
% of Input
0
2
4
6
8
Peak No.1
Peak No.2

Supplementary Fig. 3. LSD1 Binds ZFP36L1, REST, YAP1 and WWTR1.

(**a-d**) Read density tracks, visualized using cistrome.db database, of normalized ChIP-seq for LSD1 (red), H3K4me1 (blue) and H3K4me2 (green) in SH-SY5Y neuroblastoma cells. Shaded area highlights region of ZFP36L1 (**a**), REST (**b**), YAP1 (**c**) and WWTR1 (**d**) with LSD1/H3K4me1/H3K4me2 binding. (**e and f**) ChIP-qPCR of NCI-H1876 (**e**) and CORL47 (**f**) cells first treated with ORY-1001 100 nM or DMSO for 6 days before performing immunoprecipitation (IP) for LSD1 followed by qPCR of ZFP36L1 of Peak No. 1 and Peak No. 2 as indicated in a. Peak No. 1 is the peak used in Figs. 2h&i. For e,f, n=2 biological independent experiment for LSD1 IP.

Fig. S4

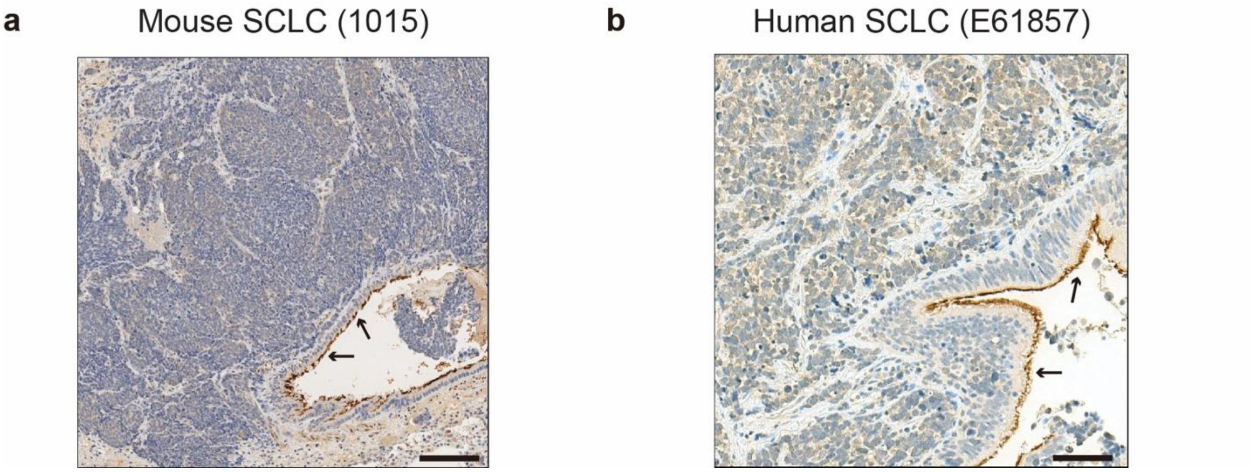

Supplementary Fig. 4. ZFP36L1 is repressed in Small Cell Lung Cancer Relative to Normal Bronchial Epithelium

(**a,b**) Representative IHC for ZFP36L1 in a mouse (**a**) and human (**b**) SCLC tumor highlighting low expression of ZFP36L1 in tumor cells with high expression in normal bronchial epithelium. For a, ZFP36L1 IHC is representative from 8 murine SCLC lung tumors from independent mice. For b, ZFP36L1 IHC is representative from 2 independent human SCLC lung tumors. Scale bar=100 μm.

Fig. S5

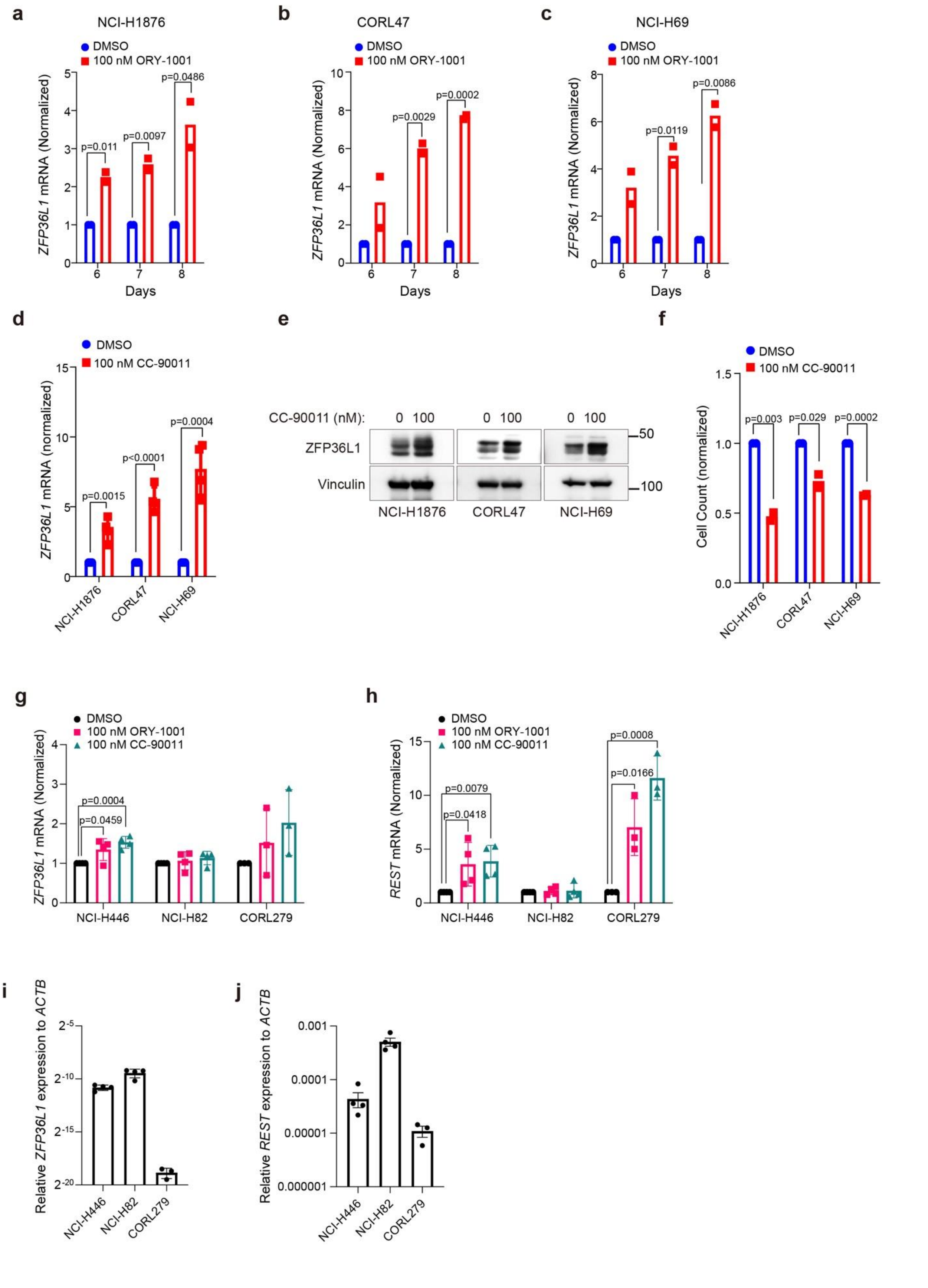
a
NCI-H1876
DMSO
100 nM ORY-1001
ZFP36L1 mRNA (Normalized)
p=0.011
p=0.0097
p=0.0486
Days
b
CORL47
DMSO
100 nM ORY-1001
ZFP36L1 mRNA (Normalized)
p=0.0029
p=0.0002
Days
c
NCI-H69
DMSO
100 nM ORY-1001
ZFP36L1 mRNA (Normalized)
p=0.0119
p=0.0086
Days
d
DMSO
100 nM CC-90011
ZFP36L1 mRNA (normalized)
p=0.0015
p<0.0001
p=0.0004
NCI-H1876
CORL47
NCI-H69
e
CC-90011 (nM): 0 100 0 100 0 100
ZFP36L1
Vinculin
50
100
NCI-H1876
CORL47
NCI-H69
f
DMSO
100 nM CC-90011
Cell Count (normalized)
p=0.003
p=0.029
p=0.0002
NCI-H1876
CORL47
NCI-H69
g
DMSO
100 nM ORY-1001
100 nM CC-90011
ZFP36L1 mRNA (Normalized)
p=0.0004
p=0.0459
NCI-H446
NCI-H82
CORL279
h
DMSO
100 nM ORY-1001
100 nM CC-90011
REST mRNA (Normalized)
p=0.0079
p=0.0418
p=0.0008
p=0.0166
NCI-H446
NCI-H82
CORL279
i
Relative ZFP36L1 expression to ACTB
NCI-H446
NCI-H82
CORL279
j
Relative REST expression to ACTB
0.001
0.0001
0.00001
0.000001
NCI-H446
NCI-H82
CORL279

Supplementary Fig. 5. ZFP36L1 is Induction by LSD1 Inhibitors in ASCL1-positive Small Cell Lung Cancer Cell Lines.

(**a-c**) RT-qPCR of NCI-H1876 (**a**), CORL47 (**b**), and NCI-H69 cells (**c**) treated with ORY-1001 (100 nM) for 6, 7 and 8 days. For a-c, n=2 biological independent experiments. (**d-f**) RT-qPCR (**d**), immunoblot analysis (**e**), and cell counts (**f**) of NCI-H1876, CORL47, and NCI-H69 cells treated with the LSD1 inhibitor CC-90011 (100 nM) for 7 days. For d, n=4 biological independent experiments. For f, n=2 biological independent experiments. (**g** and **h**) RT-qPCR for ZFP36L1 (**g**) or REST (**h**) in the NEUROD1-positive SCLC cell lines NCI-H446, NCI-H82, and CORL279 after treatment with ORY-1001 or CC-90011 for 7 days. For g and h, n=4 biological independent experiments (NCI-H446 and NCI-H82) and n=3 biological independent experiments (CORL279). **(i, j)** ΔC_T method followed by $2^{-\Delta CT}$ of the RT-qPCR data from **g,h** to determine baseline gene expression of ZFP36L1 **(i)** and *REST* **(j)** relative to *ACTB* for the small cell lung cancer cell lines indicated**.** For i and j, n=4 biological independent experiments (NCI-H446 and NCI-H82) and n=3 biological independent experiments (CORL279). For d,g-j, data are presented as mean values +/- SEM. For all panels, statistical significance was calculated using unpaired, two-tailed students t-test and p-values are indicated.

Fig. S6

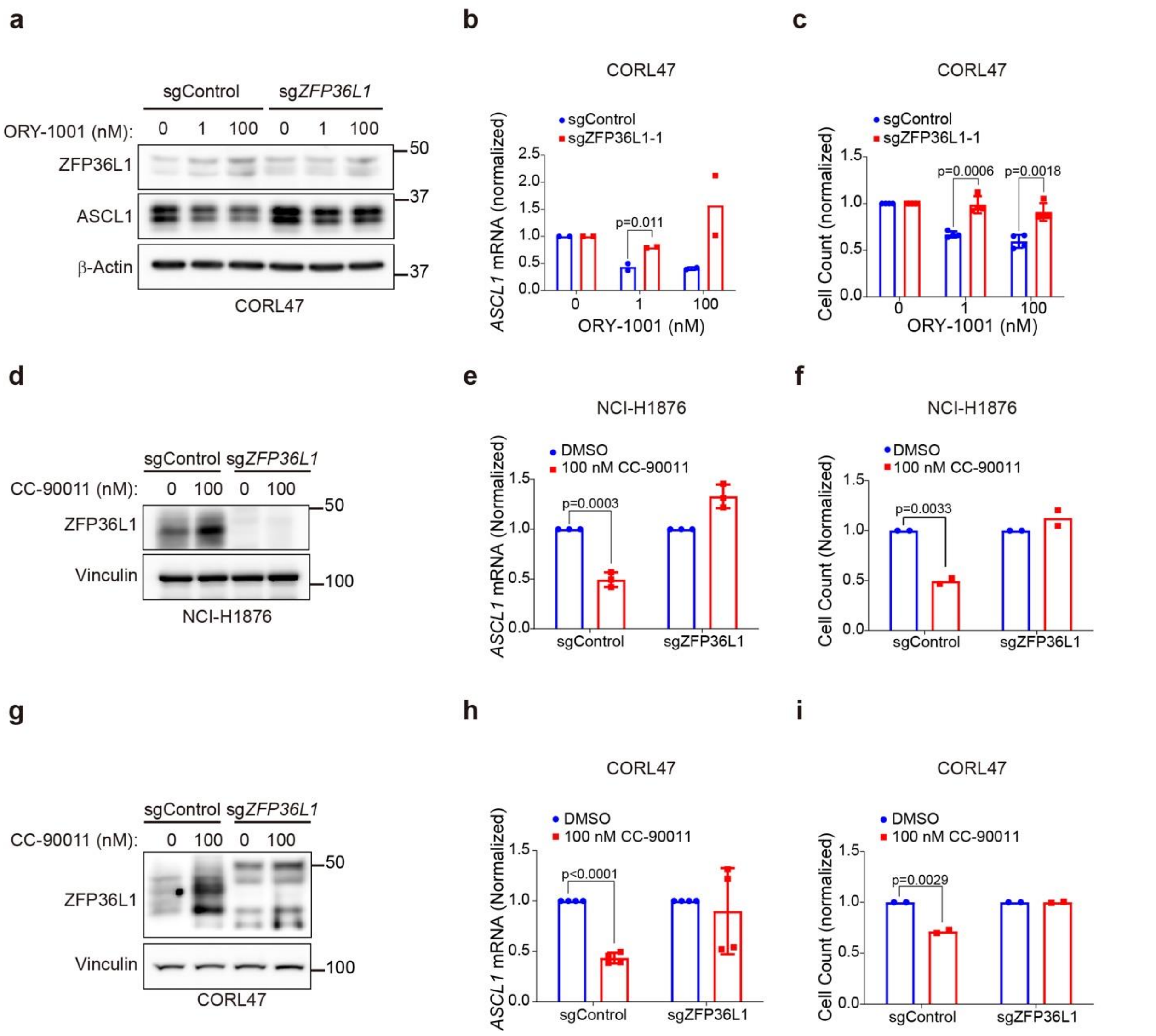

Supplementary Fig. 6. LSD1 Inhibitors Block Neuroendocrine Differentiation and Proliferation in Small Cell Lung Cancer Through a ZFP36L1-Dependent Mechanism

(**a-c**) Immunoblot analysis (**a**), RT-qPCR (**b**), and cell counts (**c**) of CORL47 Cas9 cells infected with lentiviruses encoding an sgRNA targeting ZFP36L1 (sgZFP36L1) or a non-targeting sgRNA (sgControl) and then treated with ORY-1001 (1 nM and 100 nM) or DMSO for 7 days. For b, n=2

biological independent experiments. For c, n=4 biological independent experiments. (**d-i**) Immunoblot analysis (**d and g**), RT-qPCR (**e and h**) and quantitation of cell counts (**f and i**) of NCI-H1876 Cas9 cells (**d-f**) and CORL47 Cas9 cells (**g-i**) first infected with sgZFP36L1 or sgControl lentiviruses and then treated with CC-90011 (100 nM) or DMSO for 7 days. For e, n=3 biological independent experiments. For f, n=2 biological independent experiments. For h, n=4 biological independent experiments. For i, n=2 biological independent experiments. For c,e,h, data are presented as mean values +/- SEM. For all panels, statistical significance was calculated using unpaired, two-tailed students t-test and p-values are indicated.

Fig. S7

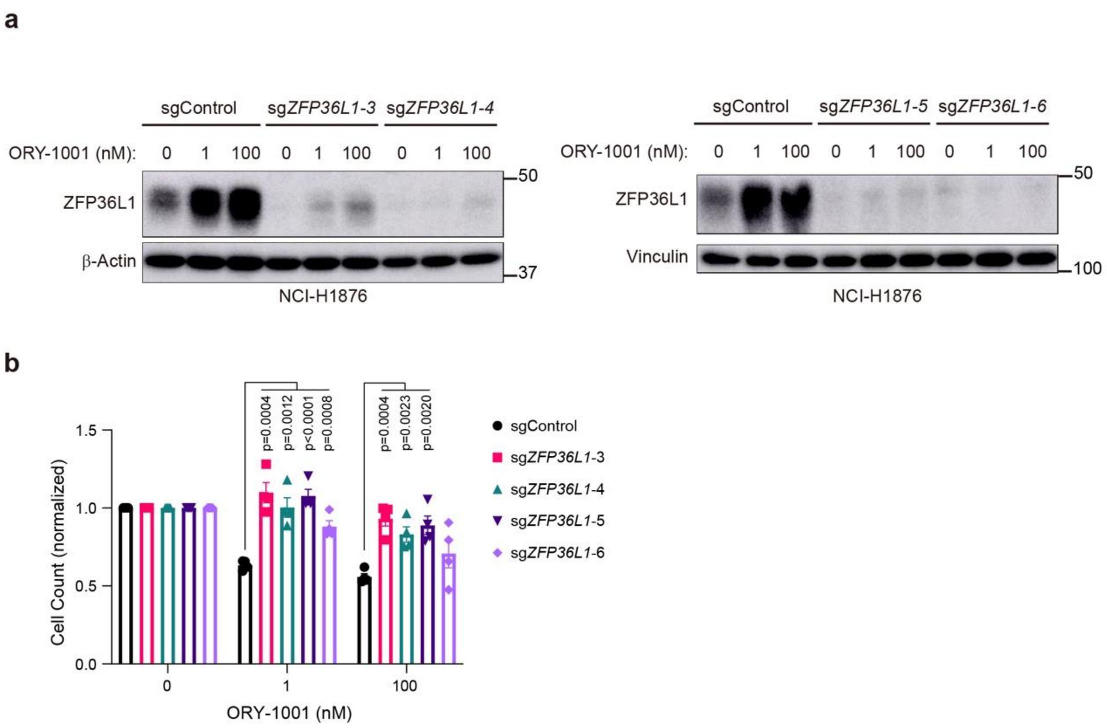

Supplementary Fig. 7. ZFP36L1 is Required for the Anti-Proliferative Effects of ORY-1001 in NCI-H1876 Cells

Immunoblot analysis (**a**) and quantitation of cell counts (**b**) of NCI-H1876 Cas9 cells infected lentiviruses encoding 4 independent sgRNAs targeting ZFP36L1 (labeled as 3-6) or a non-targeting sgRNA (sgControl) and then treated with ORY-1001 (1 nM and 100 nM) or DMSO for 7 days. For b, n=4 biological independent experiments. For b, data are presented as mean values +/- SEM. Statistical significance was calculated using unpaired, two-tailed students t-test and all ZFP36L1 sgRNAs compared to sgControl at both ORY-1001 concentrations were statistically significant except for sgZFP36L1-6 vs. sgControl at 100 nM where p=0.16. All other p-values are indicated on graph.

Fig. S8

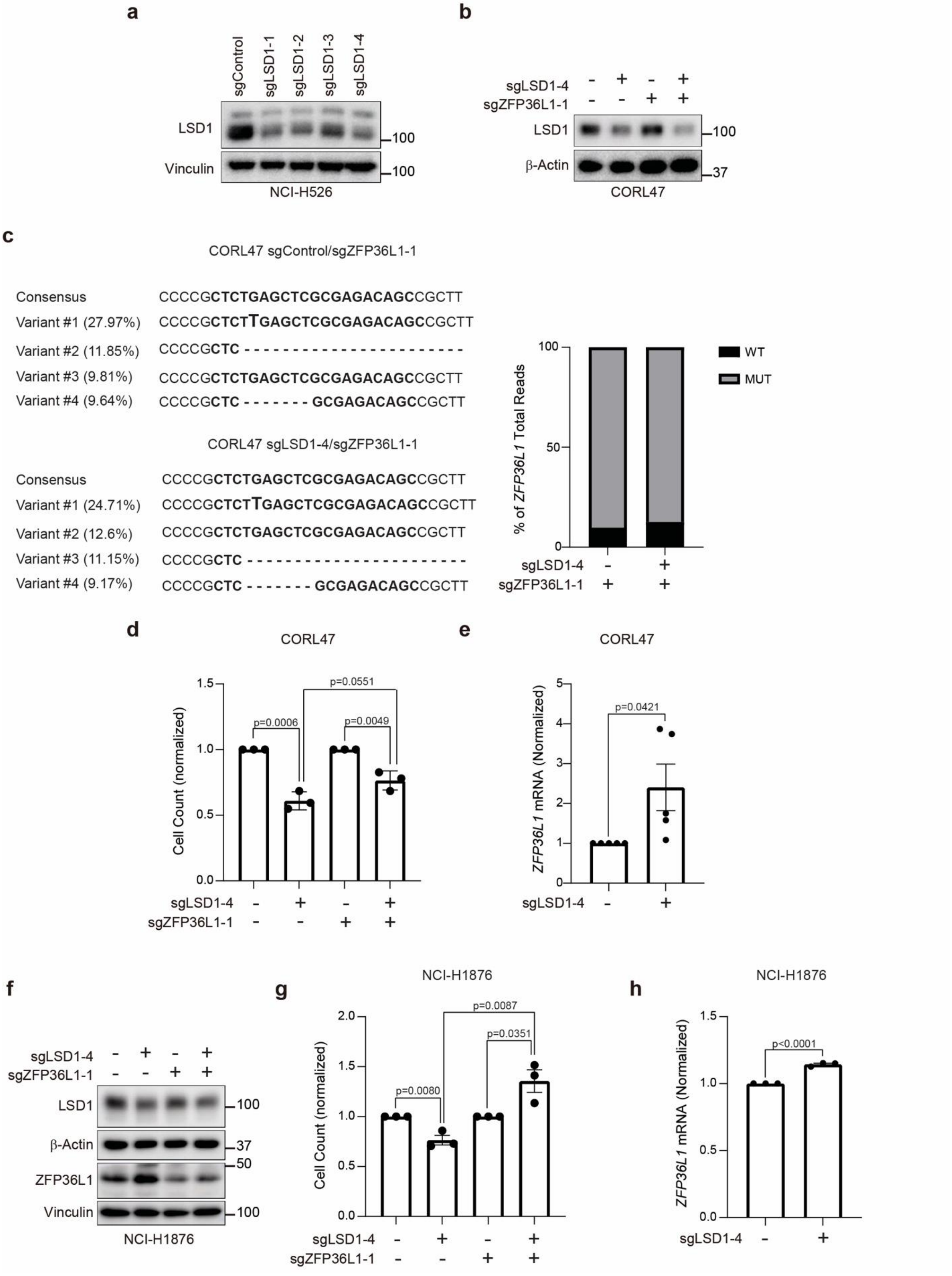

a
sgControl
sgLSD1-1
sgLSD1-2
sgLSD1-3
sgLSD1-4
LSD1
Vinculin
100
100
NCI-H526
b
sgLSD1-4
sgZFP36L1-1
LSD1
β-Actin
100
37
CORL47
c
CORL47 sgControl/sgZFP36L1-1
Consensus CCCCGCTCTGAGCTCGCGAGACAGCCGCTT
Variant #1 (27.97%) CCCCGCTCTTGAGCTCGCGAGACAGCCGCTT
Variant #2 (11.85%) CCCCGCTC -
Variant #3 (9.81%) CCCCGCTCTGAGCTCGCGAGACAGCCGCTT
Variant #4 (9.64%) CCCCGCTC - - - - - - - GCGAGACAGCCGCTT
CORL47 sgLSD1-4/sgZFP36L1-1
Consensus CCCCGCTCTGAGCTCGCGAGACAGCCGCTT
Variant #1 (24.71%) CCCCGCTCTTGAGCTCGCGAGACAGCCGCTT
Variant #2 (12.6%) CCCCGCTCTGAGCTCGCGAGACAGCCGCTT
Variant #3 (11.15%) CCCCGCTC -
Variant #4 (9.17%) CCCCGCTC - - - - - - - GCGAGACAGCCGCTT
% of ZFP36L1 Total Reads
WT
MUT
sgLSD1-4
sgZFP36L1-1
d
CORL47
Cell Count (normalized)
p=0.0006
p=0.0551
p=0.0049
sgLSD1-4
sgZFP36L1-1
e
CORL47
ZFP36L1 mRNA (Normalized)
p=0.0421
sgLSD1-4
f
sgLSD1-4
sgZFP36L1-1
LSD1
β-Actin
ZFP36L1
Vinculin
100
37
50
100
NCI-H1876
g
NCI-H1876
Cell Count (normalized)
p=0.0080
p=0.0087
p=0.0351
sgLSD1-4
sgZFP36L1-1
h
NCI-H1876
ZFP36L1 mRNA (Normalized)
p<0.0001
sgLSD1-4

Supplementary Fig. 8. LSD1 CRISPR Inactivation Phenocopies LSD1 Inhibitor Phenotypes Related to ZFP36L1.

(**a**) Immunoblot analysis of NCI-H526 Cas9 cells infected with the indicated sgRNAs. NCI-H526 were used to screen for sgRNAs that effectively knocked out LSD1 in SCLC as among SCLC cell lines, NCI-H526 cells are highly efficient at CRISPR/Cas9 editing. LSD1 sg4 was chosen for experiments below. (**b** and **f**) Immunoblot analysis of CORL47 Cas9 sgZFP36L1#1 or sgControl cells (see Supplementary Fig. 6a) (**b**) or NCI-H1876 Cas9 sgZFP36L1#1 or sgControl cells (see Fig. 4c) (**f**) that were superinfected with lentiviruses encoding an sgRNA targeting LSD1 (sgLSD1#4) or a non-targeting sgRNA (sgControl). (**c**) CRISPR amplicon sequencing for ZFP36L1 in the CORL47 ZFP36L1 knockout cells in b showing the 4 most common CRISPR-mediated insertion-deletion (indels) mutants (left) and the percent of reads with indels for ZFP36L1 for the cell lines indicated (right). (**d** and **g**) Quantitation of cell counts in the CORL47 (**d**) or NCI-H1876 (**g**) ZFP36L1/LSD1 isogenic cells from **b** and **f**, respectively. For d, cell counts were performed 12 days after plating. For g, cells counts were performed 5 days after plating. For d,g, n=3 biological independent experiments. RT-qPCR of CORL47 (**e**) or NCI-H1876 (**h**) LSD1 CRISPR isogenic cells from b and f at steady state after infection with the indicated sgRNAs. For e, n=5 biological independent experiments. For h, n=3 biological independent experiments. For d,e,g,h, data are presented as mean values +/- SEM, statistical significance was calculated using unpaired, two-tailed students t-test, and p-values are indicated on graph.

Fig. S9

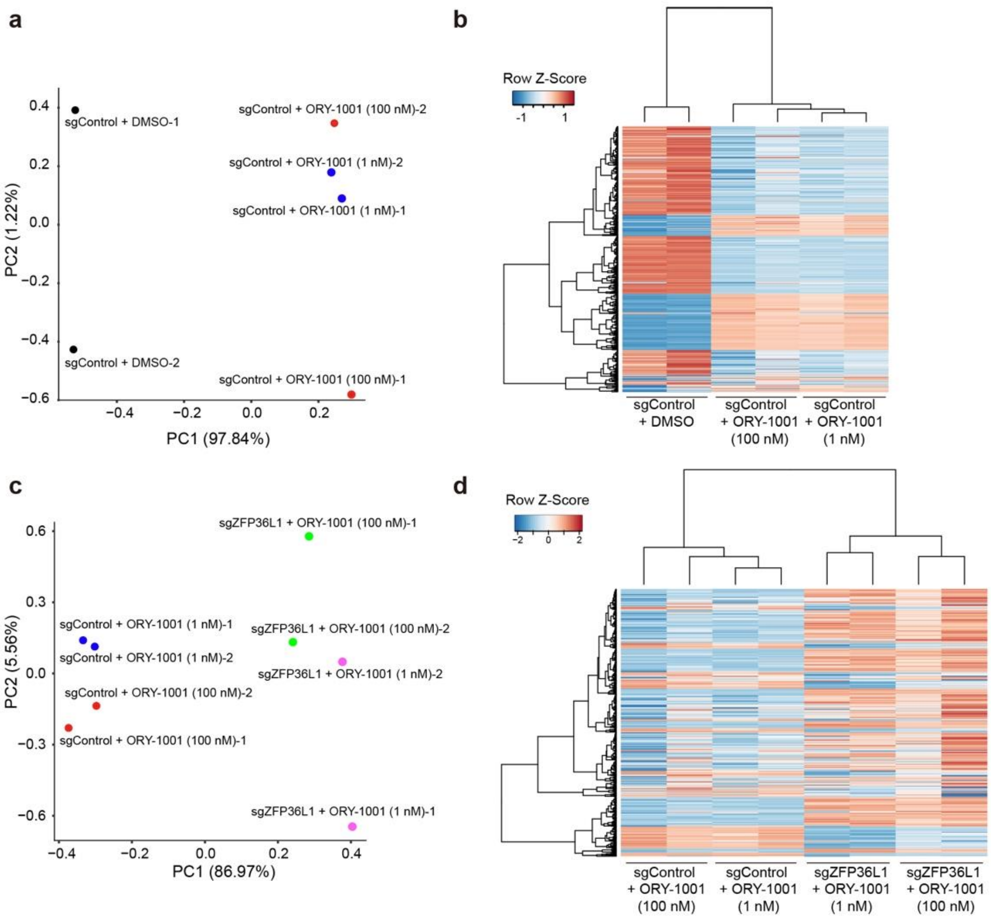

a
b
Row Z-Score
-1
1
sgControl + DMSO-1
sgControl + ORY-1001 (100 nM)-2
sgControl + ORY-1001 (1 nM)-2
sgControl + ORY-1001 (1 nM)-1
sgControl + DMSO-2
sgControl + ORY-1001 (100 nM)-1
PC2 (1.22%)
PC1 (97.84%)
0.4
0.2
0.0
−0.2
−0.4
−0.6
−0.4
−0.2
0.0
0.2
sgControl + DMSO
sgControl + ORY-1001 (100 nM)
sgControl + ORY-1001 (1 nM)
c
d
Row Z-Score
-2
0
2
sgZFP36L1 + ORY-1001 (100 nM)-1
sgControl + ORY-1001 (1 nM)-1
sgZFP36L1 + ORY-1001 (100 nM)-2
sgControl + ORY-1001 (1 nM)-2
sgZFP36L1 + ORY-1001 (1 nM)-2
sgControl + ORY-1001 (100 nM)-2
sgControl + ORY-1001 (100 nM)-1
sgZFP36L1 + ORY-1001 (1 nM)-1
PC2 (5.56%)
PC1 (86.97%)
0.6
0.3
0.0
−0.3
−0.6
−0.4
−0.2
0.0
0.2
0.4
sgControl + ORY-1001 (100 nM)
sgControl + ORY-1001 (1 nM)
sgZFP36L1 + ORY-1001 (1 nM)
sgZFP36L1 + ORY-1001 (100 nM)

Supplementary Fig. 9. RNA-Sequencing Analysis of sgZFP36L1 vs. sgControl NCI-H1876 Cells Treated with ORY-1001

(**a**) Principal component analysis (PCA) of gene expression from RNA-seq data from Fig. 4f and Supplementary Fig. 10 of NCI-H1876 sgControl cells treated with ORY-1001 (1 nM or 100 nM) or DMSO. (**b**) Unsupervised hierarchical clustering heat map of top 500 high variance genes in NCI-H1876 sgControl DMSO and sgControl ORY-1001 (1 nM and 100 nM) cells from the RNA-Seq experiment in a, Fig. 4f, and Supplementary Fig. 10. (**c**) PCA of gene expression from RNA-seq data from Fig. 4h and Supplementary Fig. 10 of NCI-H1876 sgControl cells treated with ORY-1001 (1 nM or 100 nM) or sgZFP36L1 cells treated with ORY-1001 (1 nM or 100 nM). (**d**)Unsupervised hierarchical clustering heat map of top 500 high variance genes in NCI-H1876 sgControl ORY-1001 (1 nM and 100 nM) and sgZFP36L1 ORY-1001 (1 nM and 100 nM) cells from the RNA-Seq experiment in c, Fig. 4h, and Supplementary Fig. 10. For b and d, the red to blue color scale indicates expression z-scores from large to small. For a-d, n=2 biological independent experiments for each drug condition.

Fig. S10

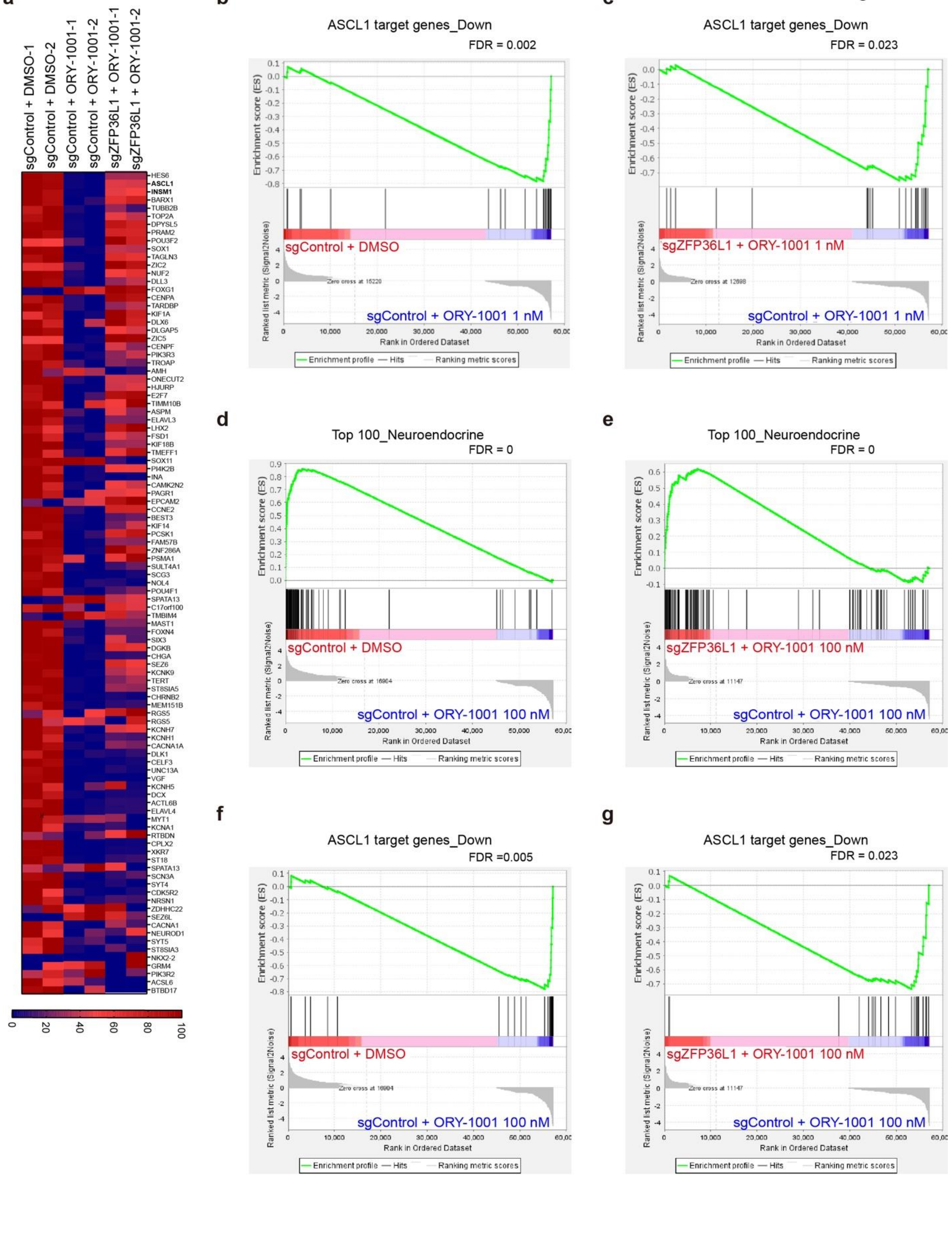
a
sgControl + DMSO-1
sgControl + DMSO-2
sgControl + ORY-1001-1
sgControl + ORY-1001-2
sgZFP36L1 + ORY-1001-1
sgZFP36L1 + ORY-1001-2
HES6
ASCL1
INSM1
BARX1
TUBB2B
TOP2A
DPYSL5
PRAM2
POU3F2
SOX1
TAGLN3
ZIC2
NUF2
DLL3
FOXG1
CENPA
TARDBP
KIF1A
DLX6
DLGAP5
ZIC5
CENPF
PIK3R3
TROAP
AMH
ONECUT2
HJURP
E2F7
TIMM10B
ASPM
ELAVL3
LHX2
FSD1
KIF18B
TMEFF1
SOX11
PI4K2B
INA
CAMK2N2
PAGR1
EPCAM2
CCNE2
BEST3
KIF14
PCSK1
FAM57B
ZNF286A
PSMA1
SULT4A1
SCG3
NOL4
POU4F1
SPATA13
C17orf100
TMBIM4
MAST1
FOXN4
SIX3
DGKB
CHGA
SEZ6
KCNK9
TERT
ST8SIA5
CHRNB2
MEM151B
RGS5
RGS5
KCNH7
KCNH1
CACNA1A
DLK1
CELF3
UNC13A
VGF
KCNH5
DCX
ACTL6B
ELAVL4
MYT1
KCNA1
RTBDN
CPLX2
XKR7
ST18
SPATA13
SCN3A
SYT4
CDK5R2
NRSN1
ZDHHC22
SEZ6L
CACNA1
NEUROD1
SYT5
ST8SIA3
NKX2-2
GRM4
PIK3R2
ACSL6
BTBD17
0
20
40
60
80
100
b
ASCL1 target genes_Down
FDR = 0.002
Enrichment score (ES)
sgControl + DMSO
Zero cross at 15220
sgControl + ORY-1001 1 nM
Ranked list metric (Signal2Noise)
Rank in Ordered Dataset
Enrichment profile — Hits — Ranking metric scores
c
ASCL1 target genes_Down
FDR = 0.023
Enrichment score (ES)
sgZFP36L1 + ORY-1001 1 nM
Zero cross at 12698
sgControl + ORY-1001 1 nM
Ranked list metric (Signal2Noise)
Rank in Ordered Dataset
Enrichment profile — Hits — Ranking metric scores
d
Top 100_Neuroendocrine
FDR = 0
Enrichment score (ES)
sgControl + DMSO
Zero cross at 16904
sgControl + ORY-1001 100 nM
Ranked list metric (Signal2Noise)
Rank in Ordered Dataset
Enrichment profile — Hits — Ranking metric scores
e
Top 100_Neuroendocrine
FDR = 0
Enrichment score (ES)
sgZFP36L1 + ORY-1001 100 nM
Zero cross at 11147
sgControl + ORY-1001 100 nM
Ranked list metric (Signal2Noise)
Rank in Ordered Dataset
Enrichment profile — Hits — Ranking metric scores
f
ASCL1 target genes_Down
FDR =0.005
Enrichment score (ES)
sgControl + DMSO
Zero cross at 16904
sgControl + ORY-1001 100 nM
Ranked list metric (Signal2Noise)
Rank in Ordered Dataset
Enrichment profile — Hits — Ranking metric scores
g
ASCL1 target genes_Down
FDR = 0.023
Enrichment score (ES)
sgZFP36L1 + ORY-1001 100 nM
Zero cross at 11147
sgControl + ORY-1001 100 nM
Ranked list metric (Signal2Noise)
Rank in Ordered Dataset
Enrichment profile — Hits — Ranking metric scores

Supplementary Fig. 10. Gene Set Enrichment Analysis of RNA-Seq Data of sgZFP36L1 vs. sgControl NCI-H1876 Cells Treated with ORY-1001

(**a**) Heatmap of the changes in the Top 100 Neuroendocrine Genes from the RNA-seq experiment in Fig. 4f-i of NCI-H1876 cells with the perturbations indicated. Red denotes genes with high expression, and blue denotes genes with low expression. (**b and c**) Gene set enrichment analysis (GSEA) of RNA-seq data in Fig. 4f (**b**) or 4h (**c**) of the ASCL1 Target Genes Down gene set. (**d-g)** GSEA for the Top 100 Neuroendocrine Genes (**d, e**) or ASCL1 Target Genes Down (**f, g**) of RNA-seq data from NCI-H1876 sgZFP36L1 or sgControl cells treated with 100 nM ORY-1001 or DMSO as indicated. FDR q-values are indicated. For b-g, n=2 biological independent experiments for each drug condition.

Fig. S11

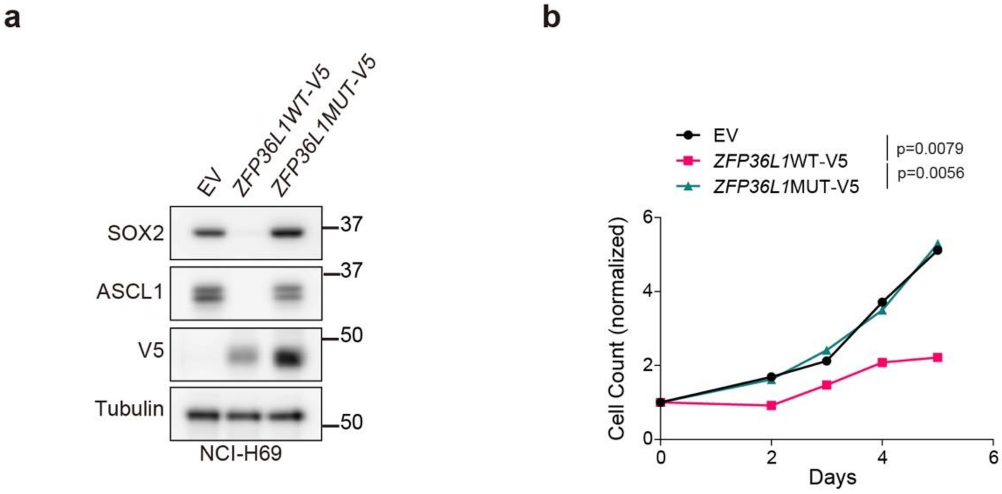

Supplementary Fig. 11. The mRNA-binding activity of ZFP36L1 is required to block neuroendocrine differentiation and cellular proliferation in NCI-H69 Cells.

(**a**) Immunoblot analysis of NCI-H69 cells stably infected with ZFP36L1WT-V5, the ZFP36L1 mRNA-binding mutant-V5 (ZFP36L1MUT-V5), or the corresponding empty vector (EV). (**b**) Proliferation assays of the cells in A. n=2 biological independent experiments. Statistical significance was calculated using unpaired, two-tailed students t-test and p-values are indicated.

Fig. S12

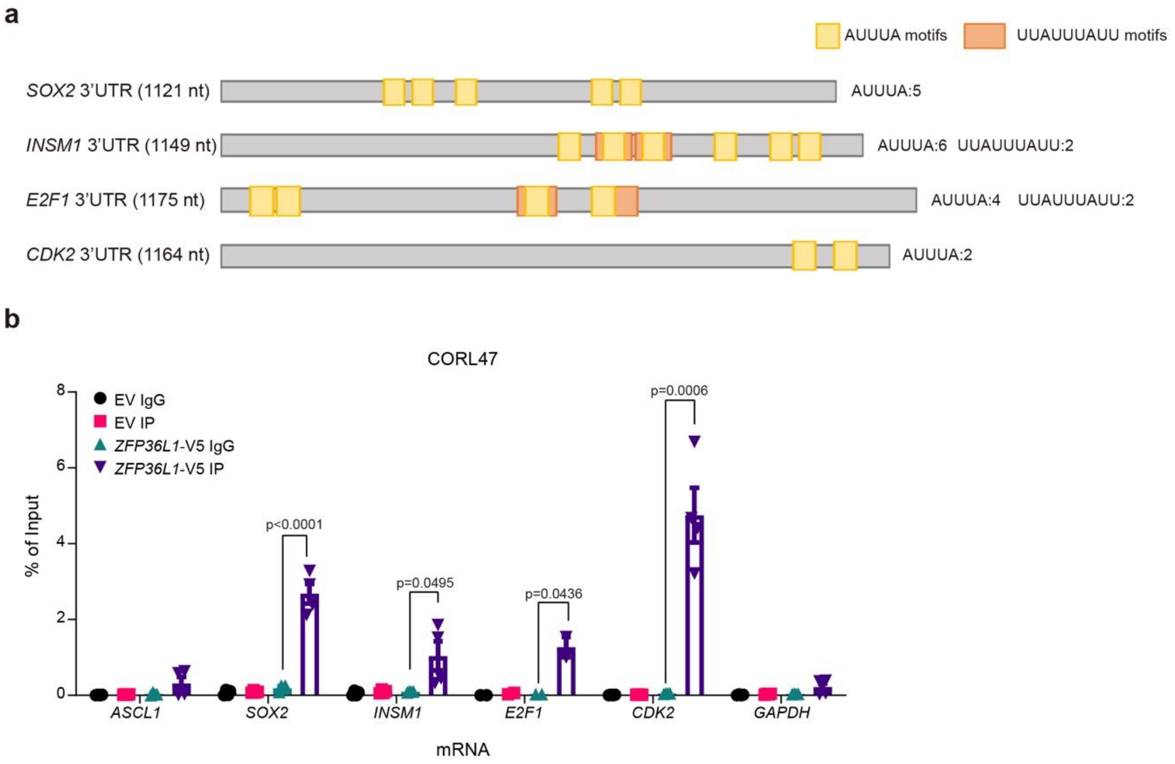

Supplementary Fig. 12. ZFP36L1 Binds SOX2, INSM1, E2F1, and CDK2 mRNAs.

(**a**) Schematic of the ZFP36L1 canonical binding sites (AU-rich elements=AREs) in the 3'UTR's of SOX2, INSM1, E2F1, and CDK2. The number of ARE's are indicated. Yellow is AUUUA motifs and orange is UUAUUUAUU motifs. (**b**) mRNA quantitation relative to input after RT-qPCR from immunoprecipitation (IP) of ZFP36L1WT-V5, ZFP36L1MUT-V5, or EV from CORL47 cells relative to input with primers specific to ASCL1, SOX2, INSM1, E2F1, CDK2, and GAPDH. n=4 biological independent experiments for all genes except E2F1 where n=2 biological independent experiments. For c, data are presented as mean values +/- SEM, statistical significance was calculated using unpaired, two-tailed students t-test, and p-values are indicated.

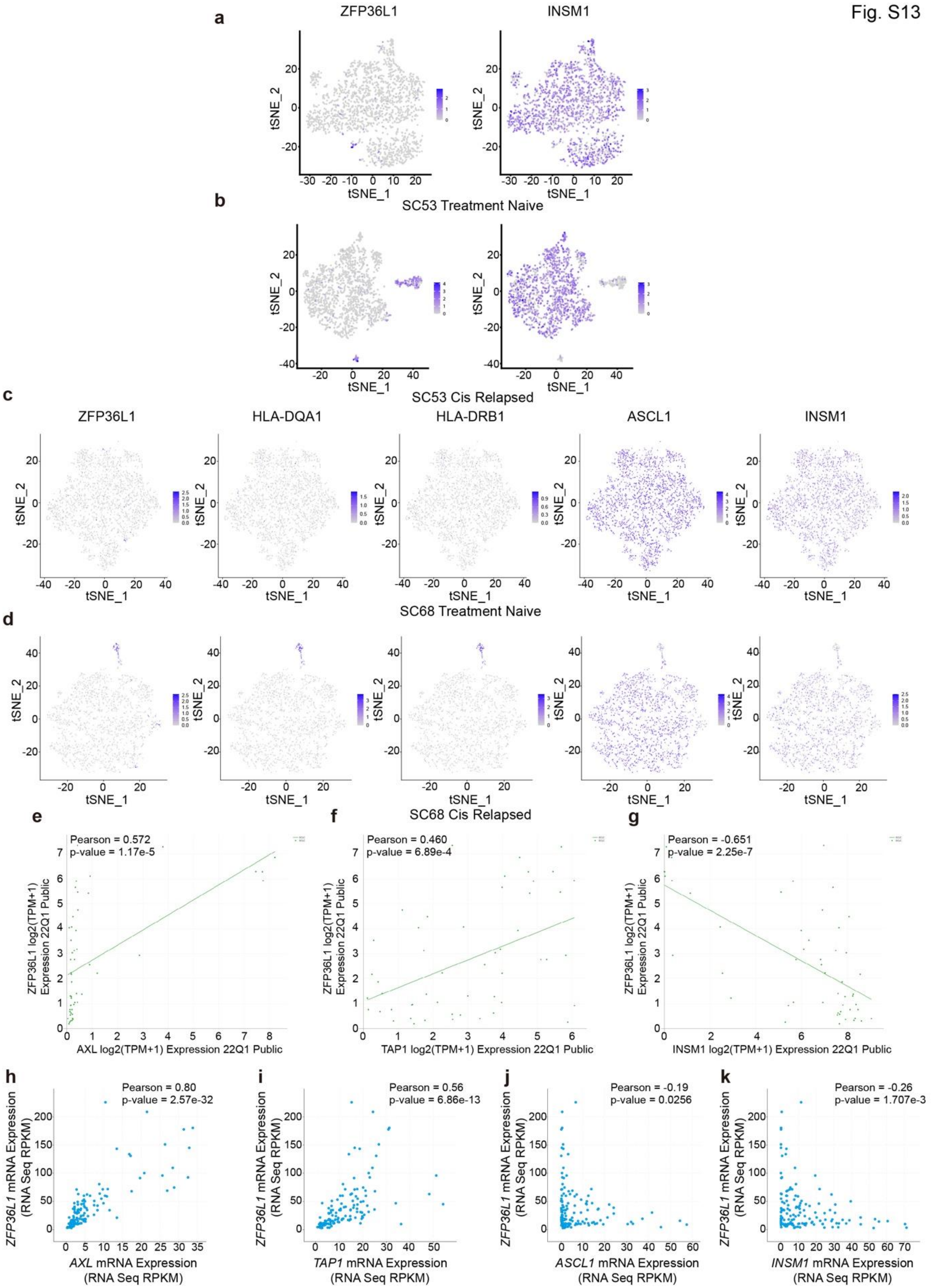

Fig. S13
a
ZFP36L1
INSM1
tSNE_2
tSNE_1
SC53 Treatment Naive
b
SC53 Cis Relapsed
c
ZFP36L1
HLA-DQA1
HLA-DRB1
ASCL1
INSM1
SC68 Treatment Naive
d
SC68 Cis Relapsed
e
Pearson = 0.572
p-value = 1.17e-5
ZFP36L1 log2(TPM+1) Expression 22Q1 Public
AXL log2(TPM+1) Expression 22Q1 Public
f
Pearson = 0.460
p-value = 6.89e-4
TAP1 log2(TPM+1) Expression 22Q1 Public
g
Pearson = -0.651
p-value = 2.25e-7
INSM1 log2(TPM+1) Expression 22Q1 Public
h
Pearson = 0.80
p-value = 2.57e-32
ZFP36L1 mRNA Expression (RNA Seq RPKM)
AXL mRNA Expression (RNA Seq RPKM)
i
Pearson = 0.56
p-value = 6.86e-13
TAP1 mRNA Expression (RNA Seq RPKM)
j
Pearson = -0.19
p-value = 0.0256
ASCL1 mRNA Expression (RNA Seq RPKM)
k
Pearson = -0.26
p-value = 1.707e-3
INSM1 mRNA Expression (RNA Seq RPKM)

Supplementary Fig. 13. ZFP36L1 is Correlates with Markers of the Inflammatory Subtype of Small Cell Lung Cancer.

(a and b) t-SNE plots of single tumor cells from SC53 treatment naïve (**a**) and after cisplatin relapse (**b**) showing ZFP36L1 and INSM1 expression. (**c and d**) t-SNE plots of single tumor cells from SC68 treatment naïve (**c**) and after cisplatin relapse (**d**) showing ZFP36L1, HLA-DQA1, HLA-DRB1, ASCL1, and INSM1 expression. (**e-g**) Correlation analysis *ZFP36L1* vs. *AXL* (**e**), *TAP1* (**f**), and *INSM1* (**g**) using publicly available RNA-sequencing data of all SCLC cell lines from the CCLE. Pearson correlation coefficients and p-values are indicated. (**h-k**) Correlation analysis *ZFP36L1* vs. *AXL* (**h**), *TAP1* (**i**), *ASCL1* (**j**), *INSM1* (**k**) mRNA expression using publicly available RNA-sequencing data (cBioPortal) from 141 human neuroblastoma tumor samples. Pearson correlation coefficients and p-values are indicated on figure panel.

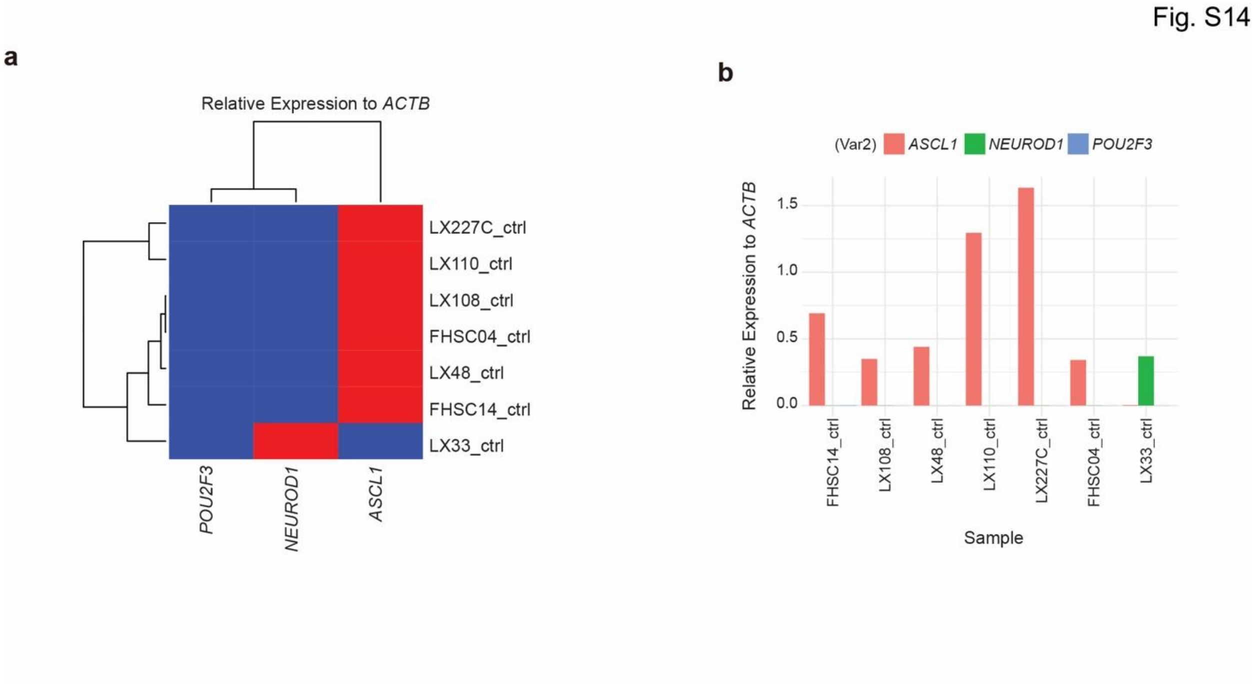

Supplementary Fig. 14. Molecular Subtypes of SCLC Patient-Derived Xenograft Models.

(a) Heatmap of molecular subtype expression of *ASCL1*, *NEUROD1*, and *POU2F3* from RNA-seq from the 7 patient-derived xenograft (PDX) models indicated normalized by row. Blue indicates low expression and red indicates high expression. **(b)** Barplot of mRNA expression of *ASCL1*, *NEUROD1*, and *POU2F3* relative to *ACTB* from RNA-seq data from the 7 SCLC PDX models indicated.

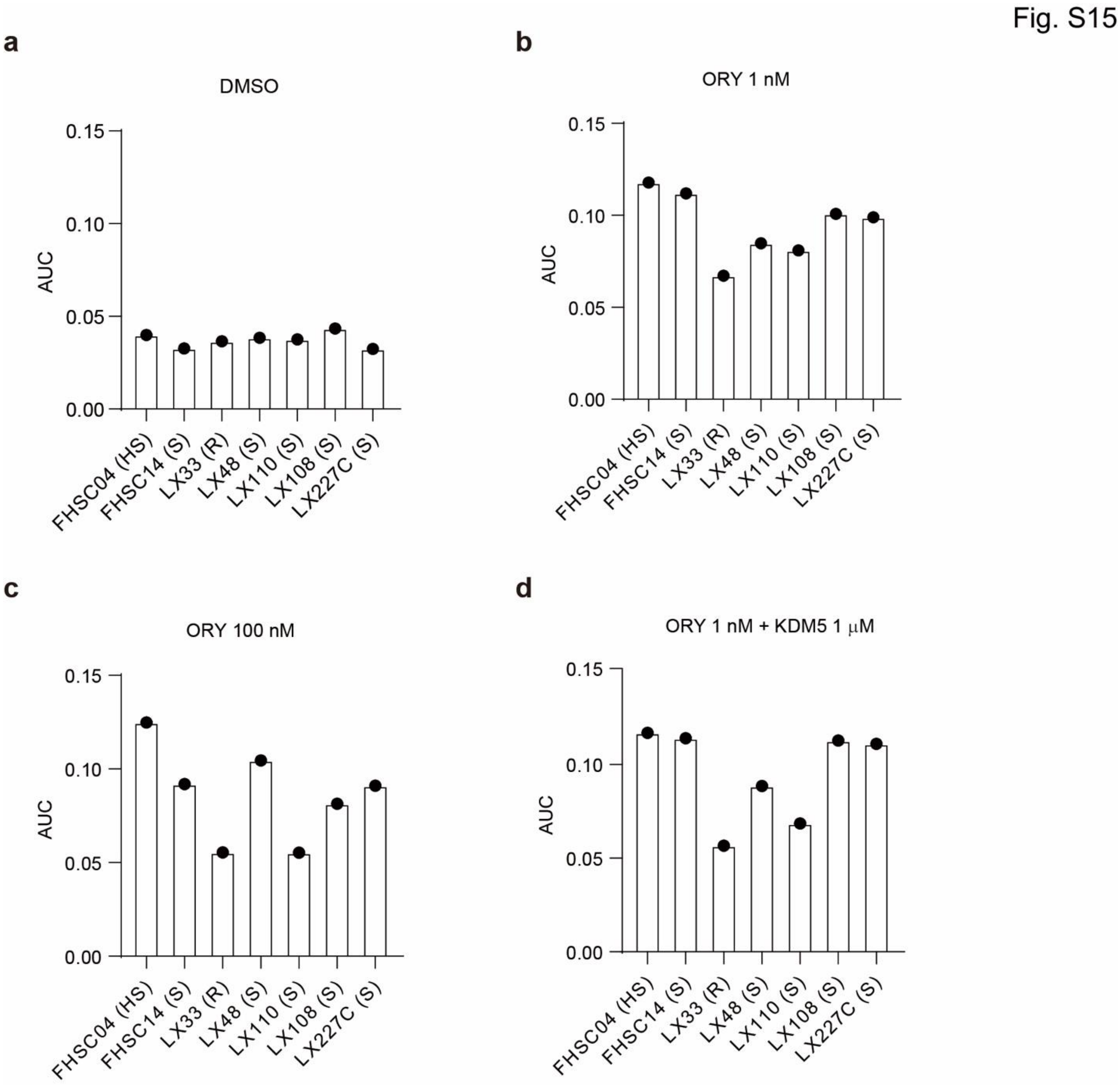

Supplementary Fig. 15. Enrichment Analysis of Correlation of Expression of Hits from our CRISPR/Cas9 ORY-1001/KDM5-C70 Resistance Screen with ORY-1001 Sensitivity.

(**a-d**) Area under the curve (AUC) enrichment analysis of hits from our ORY-1001/KDM5-C70 resistance screen (see Fig. 1c-f) of RNA-seq data from untreated patient-derived xenograft (PDX) models of SCLC. Hits were considered significant and were included in the AUC enrichment analysis if their q-value was less than 0.25 for ORY-1001 1 nM (**b**), ORY-1001 100 nM (**c**), and

ORY-1001 (1 nM) + KDM5-C70 (1000 nM) (**d**). Hits were considered significant in the DMSO arm with p-values less than 0.05. A less stringent cut-off was used in the DMSO arm because there were very few hits with q-value less than 0.25 in the DMSO arm. Note that no relative AUC enrichment was observed between PDX models in the DMSO arm.

Fig. S16

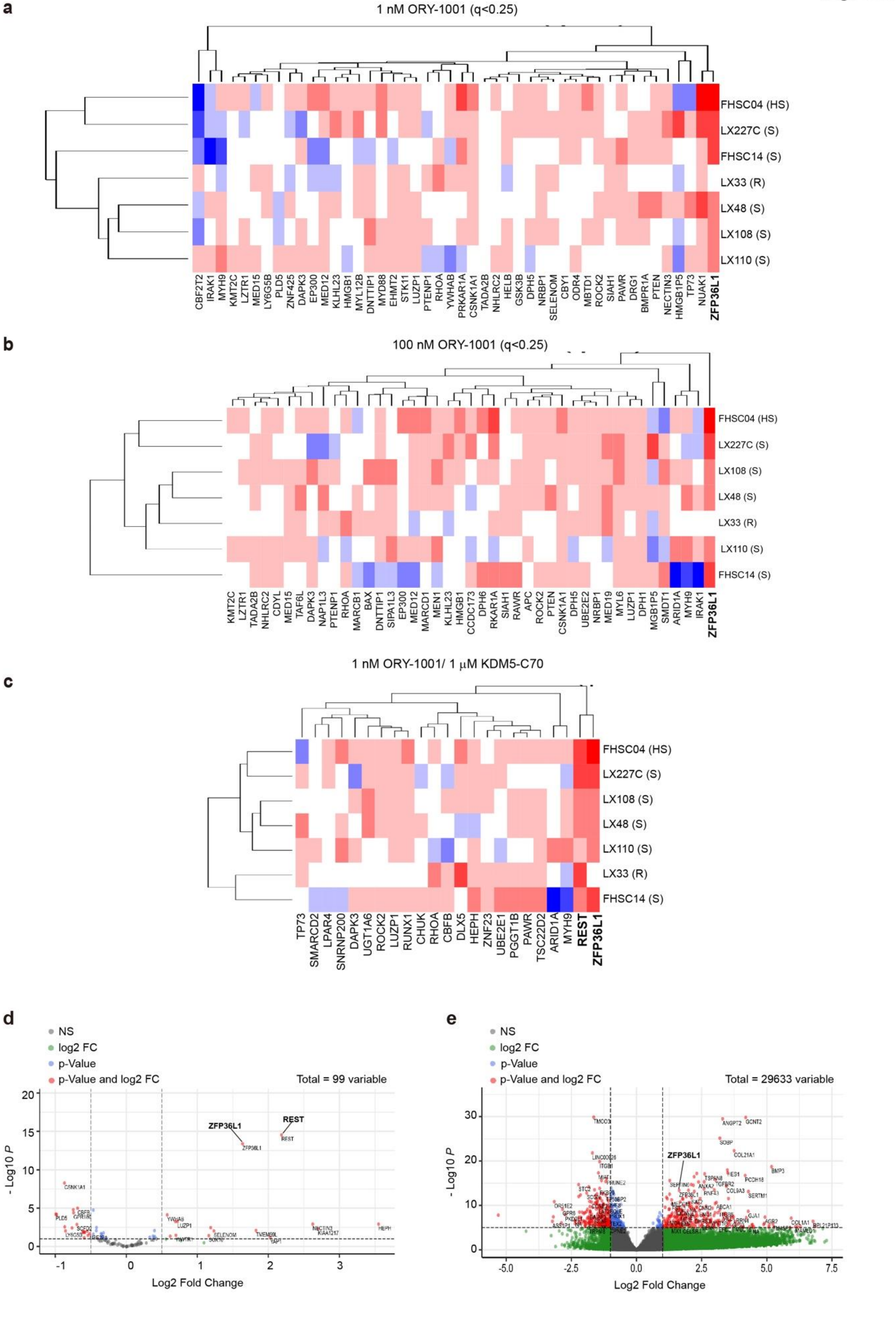
a
1 nM ORY-1001 (q<0.25)
FHSC04 (HS)
LX227C (S)
FHSC14 (S)
LX33 (R)
LX48 (S)
LX108 (S)
LX110 (S)
CBF2T2 IRAK1 MYH9 KMT2C LZTR1 MED15 LY6G5B PLD5 ZNF425 DAPK3 EP300 MED12 KLHL23 HMGB1 MYL12B DNTTIP1 MYD88 EHMT2 STK11 LUZP1 PTENP1 RHOA YWHAB PRKAR1A CSNK1A1 TADA2B NHLRC2 HELB GSK3B DPH5 NRBP1 SELENOM CBY1 ODR4 MBTD1 ROCK2 SIAH1 PAWR DRG1 BMPR1A PTEN NECTIN3 HMGB1P5 TP73 NUAK1 ZFP36L1
b
100 nM ORY-1001 (q<0.25)
FHSC04 (HS)
LX227C (S)
LX108 (S)
LX48 (S)
LX33 (R)
LX110 (S)
FHSC14 (S)
KMT2C LZTR1 TADA2B NHLRC2 CDYL MED15 TAF6L DAPK3 NAP1L3 PTENP1 RHOA MARCB1 BAX DNTTIP1 SIPA1L3 EP300 MED12 MARCD1 MEN1 KLHL23 HMGB1 CCDC173 DPH6 RKAR1A SIAH1 RAWR APC ROCK2 PTEN CSNK1A1 DPH5 UBE2E2 NRBP1 MED19 MYL6 LUZP1 DPH1 MGB1P5 SMDT1 ARID1A MYH9 IRAK1 ZFP36L1
c
1 nM ORY-1001/ 1 μM KDM5-C70
FHSC04 (HS)
LX227C (S)
LX108 (S)
LX48 (S)
LX110 (S)
LX33 (R)
FHSC14 (S)
TP73 SMARCD2 LPAR4 SNRNP200 DAPK3 UGT1A6 ROCK2 LUZP1 RUNX1 CHUK RHOA CBFB DLX5 HEPH ZNF23 UBE2E1 PGGT1B PAWR TSC22D2 ARID1A MYH9 REST ZFP36L1
d
NS
log2 FC
p-Value
p-Value and log2 FC
Total = 99 variable
ZFP36L1
REST
- Log10 P
Log2 Fold Change
e
NS
log2 FC
p-Value
p-Value and log2 FC
Total = 29633 variable
ZFP36L1
- Log10 P
Log2 Fold Change

Supplementary Fig. 16. Unsupervised Hierarchical Clustering of Hits from our CRISPR/Cas9 ORY-1001/KDM5-C70 Resistance Screen after ORY-1001 Treatment.

(**a-c**) Unsupervised hierarchical clustering of hits from our ORY-1001 1 nM (**a**), ORY-1001 100 nM (**b**), and ORY-1001 (1 nM) + KDM5-C70 (1000 nM) (**C**) CRISPR/Cas9 resistance screen (see Figs. 1C-E) of RNA-seq data from SCLC PDX models treated ex-vivo with ORY-1001 relative to DMSO. Red denotes genes with high expression, and blue denotes genes with low expression. (**d and e**) Volcano plot of RNA-sequencing (RNA-seq) data from the highly sensitive SCLC PDX model FHSC04 treated with ORY-1001 or vehicle *in vivo* showing the log2 fold change and -log10 p-value of gene expression of hits from our ORY-1001 (100 nM) + KDM5-C70 (1000 nM) CRISPR/Cas9 ORY-1001 resistance screen with q-values less than 0.25 (**d**) or all mRNAs (**e**). Note that similar to the *ex vivo* results observed in Fig. 7b and Supplementary Figs. 16a-c, ZFP36L1 and REST are most statistically significantly induced *in vivo*.

Fig. S17

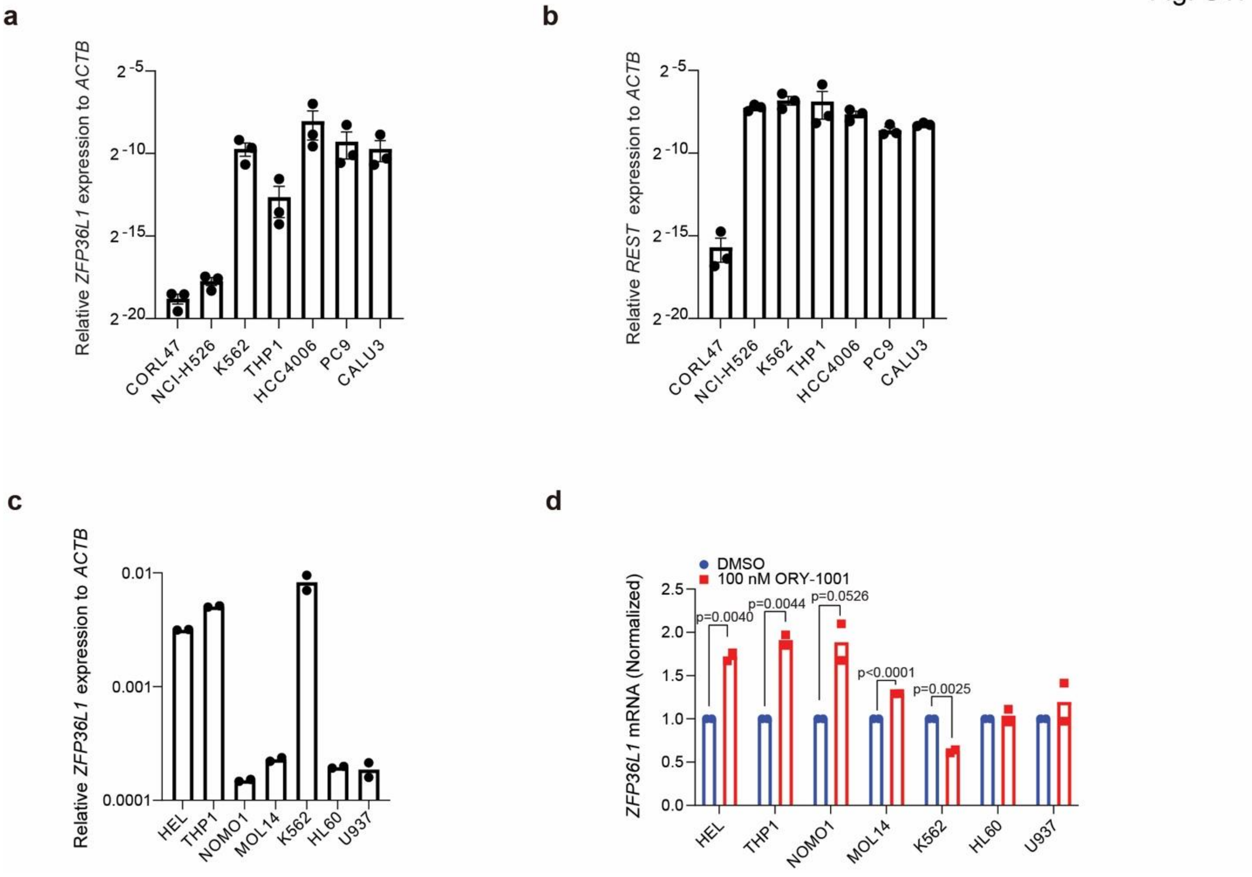

Supplementary Fig. 17. ΔC_T Values for Baseline Gene Expression in Leukemia Cell Lines Used in this Study.

(**a,b**) ΔC_T method followed by $2^{-\Delta CT}$ to determine baseline gene expression of *ZFP36L1* **(a),** *REST* **(b)** for the cancer cell lines indicated from the RT-qPCR data in fig. 7d,e, respectively. For a,b, n=3 biological independent experiments and data are presented as mean values +/- SEM. **(c)** ΔC_T method followed by $2^{-\Delta CT}$ to determine baseline gene expression of ZFP36L1 relative to *ACTB* for the leukemia cell lines indicated**.** n=2 biological independent experiments. **(d)** RT-qPCR for ZFP36L1 of the leukemia cell lines indicated treated with ORY-1001 (100 nM) or DMSO. n=2 biological independent experiments. Statistical significance was calculated using unpaired, two-tailed students t-test and p-values are indicated on figure.
