# Supplementary material for: Regulation of neuroendocrine plasticity by the RNA-binding protein ZFP36L1
Source: Nat Commun. 2022 Aug 25;13:4998. doi: 10.1038/s41467-022-31998-7 (PMC9411550; doi:10.1038/s41467-022-31998-7)
Supplement: Supplementary file 3 — Description of Additional Supplementary Files [file 41467_2022_31998_MOESM3_ESM.pdf]

Description of Additional Supplementary Files

File Name: Supplementary Data 1
Description: **CRISPR/Cas9 ORY-1001 and ORY-1001/KDM5-C70 Inhibitor Resistance Screening Data.** Tab 1 contains Log_2 normalized scores under the specified drug conditions at day 35 with the early timepoint (day 11) subtracted. Tabs 2-5 contain the STARS analysis under the specified drug conditions. Tabs 6-9 contains the Hypergeometric analysis under the specified conditions.

File Name: Supplementary Data 2
Description: **RNA-Sequencing Data of NCI-H1876 sgZFP36L1 (ZFP36L1-KO) or sgControl (ZFP36L1-WT) Cells Treated with ORY-1001 or DMSO.** (Tab 1) FPKM values of RNA-sequencing data of the indicated isogenic ZFP36L1 NCI-H1876 cell lines treated with ORY-1001 (1 nM) or DMSO for 7 days. n=2 biological replicates. (Tab 2) Differential expression analysis of RNA-seq data from tab 1 of NCI-H1876 sgControl cells treated with ORY-1001 (1 nM) compared to NCI-H1876 sgControl cells treated with DMSO. (Tab 3) Differential expression analysis of RNA-seq data from tab 1 of NCI-H1876 sgZFP36L1 cells treated with ORY-1001 (1 nM) compared to NCI-H1876 sgControl cells treated with ORY-1001 (1 nM). (Tab 4) Differential expression analysis of RNA-seq data from tab 1 of NCI-H1876 sgControl cells treated with ORY-1001 (100 nM) compared to NCI-H1876 sgControl cells treated with DMSO. (Tab 5) Differential expression analysis of RNA-seq data from tab 1 of NCI-H1876 sgZFP36L1 cells treated with ORY-1001 (100 nM) compared to NCI-H1876 sgControl cells treated with ORY-1001 (100 nM).

File Name: Supplementary Data 3
Description: **RNA-Sequencing Data of CORL47 dCas9-VP64 Cells Infected with 2 Independent sgRNAs to Activate Endogenous ZFP36L1.** (Tab 1) FPKM values of RNA-sequencing data of the indicated isogenic ZFP36L1 CORL47 cell lines generated using CORL47 dCas9-VP64 cells stably engineered to express sgRNAs targeting the promoter of ZFP36L1 [sgZFP36L1-1 or sgZFP36L1-2] to activate endogenous ZFP36L1 expression or a non-targeting sgRNA (sgControl). n=2 biological replicates. (Tab 2) Differential expression analysis of RNA-seq data from tab 1 of CORL47 dCas9-VP64 cells stably expressing sgZFP36L1-1 to activate endogenous ZFP36L1 compared to CORL47 dCas9-VP64 cells expressing a non-targeting control. (Tab 3) Differential expression analysis of RNA-seq data from tab 1 of CORL47 dCas9-VP64 cells stably expressing sgZFP36L1-2 to activate endogenous ZFP36L1 compared to CORL47 dCas9-VP64 cells expressing a non-targeting control.
