# Supplementary material for: Regulation of neuroendocrine plasticity by the RNA-binding protein ZFP36L1
Source: Nat Commun. 2022 Aug 25;13:4998. doi: 10.1038/s41467-022-31998-7 (PMC9411550; doi:10.1038/s41467-022-31998-7)
Supplement: Supplementary file 7 — Reporting Summary [file 41467_2022_31998_MOESM7_ESM.pdf]

nature portfolio

Corresponding author(s): Matthew G. Oser MD, PhD

Last updated by author(s): 06-29-22

Reporting Summary

Nature Portfolio wishes to improve the reproducibility of the work that we publish. This form provides structure for consistency and transparency in reporting. For further information on Nature Portfolio policies, see our Editorial Policies and the Editorial Policy Checklist.

Statistics

For all statistical analyses, confirm that the following items are present in the figure legend, table legend, main text, or Methods section.

n/a	Confirmed	
☐	☒	The exact sample size (*n*) for each experimental group/condition, given as a discrete number and unit of measurement
☐	☒	A statement on whether measurements were taken from distinct samples or whether the same sample was measured repeatedly
☐	☒	The statistical test(s) used AND whether they are one- or two-sided<br>*Only common tests should be described solely by name; describe more complex techniques in the Methods section.*
☒	☐	A description of all covariates tested
☐	☒	A description of any assumptions or corrections, such as tests of normality and adjustment for multiple comparisons
☐	☒	A full description of the statistical parameters including central tendency (e.g. means) or other basic estimates (e.g. regression coefficient) AND variation (e.g. standard deviation) or associated estimates of uncertainty (e.g. confidence intervals)
☐	☒	For null hypothesis testing, the test statistic (e.g. *F*, *t*, *r*) with confidence intervals, effect sizes, degrees of freedom and *P* value noted<br>*Give P values as exact values whenever suitable.*
☒	☐	For Bayesian analysis, information on the choice of priors and Markov chain Monte Carlo settings
☒	☐	For hierarchical and complex designs, identification of the appropriate level for tests and full reporting of outcomes
☒	☐	Estimates of effect sizes (e.g. Cohen's *d*, Pearson's *r*), indicating how they were calculated

Our web collection on statistics for biologists contains articles on many of the points above.

Software and code

Policy information about availability of computer code

Data collection

R software. Version 4.0.2.

Data analysis

RNA-Sequencing Analysis
For Gene Set Enrichment Analysis (GSEA), software was downloaded from the Gene Set Enrichment Analysis website [http://www.broad.mit.edu/gsea/downloads.jsp]. GSEA was performed using the "TOP 100 Neuroendocrine Gene Set"(Balanis, Sheu et al. 2019) or "ASCL1 Target Genes DOWN"(Osada, Tomida et al. 2008) or Hallmarks Gene Sets. Gene Sets with an FDR q-value<0.25 were considered significant. For principal component analysis and cluster analysis, top 500 genes in terms of the largest standard deviation were subjected to principal component analysis using the prcomp function of R software (version 4.0.2). Clustering and heatmap were generated using the heatmap.2 function in the gplots package of R software.

Analysis of Publicly Available RNA-Sequencing Data
To determine the SCLC molecular subtypes of the cell lines used in our study (see Supplementary Fig. 2a), gene expression TPM values of ASCL1, NEUROD1, and POU2F3 were obtained from Broad Institute Cancer Cell Line Encyclopedia (CCLE). Values are inferred from RNA-seq data using the RSEM tool and are reported after log2 transformation, using a pseudo-count of 1; log2(TPM+1). For the RNA expression in cancer cell lines in Fig. 3a, the RNA-sequencing data was downloaded from CCLE. To identify potential binding sites for LSD1 in target genes (as shown in Supplementary Fig. 3), the Cistrome Data Browser was used.
For the RNA expression of ZFP36L1 in human lung tumors in Fig. 3b, the 81 SCLC RNA-Seq data was previously published(George, Lim et al. 2015) and publicly available on the cBioPortal(Gao, Aksoy et al. 2013). The 747 lung adenocarcinoma samples were from TCGA and publicly available on the cBioPortal(Gao, Aksoy et al. 2013). The ZFP36L1 RNA expression for each sample was normalized to the ACTB RNA for that sample. The MHC class I high vs. low human SCLC RNA-sequencing data set was previously described(Mahadevan, Knelson et al. 2021). For the correlation analysis of SCLC cell lines in Supplementary Figs. 13e-g, correlations in SCLC cell lines between ZFP36L1 and the AXL, TAP1, or INSM1 were calculated using demap.org. For the correlation analysis of human neuroblastoma tumors in Supplementary Figs. 13h-k, correlations between ZFP36L1 and AXL, TAP1, ASCL1, or INSM1 were calculated using 141 human neuroblastoma from cBioPortal(Gao, Aksoy

et al. 2013).
For the analysis of RNA-Seq data from SCLC PDX models, a previously published RNA-sequencing data set, where SCLC PDX models were treated in vivo and ex vivo with ORY-1001 or the vehicle control, was used(Augert, Eastwood et al. 2019). The RNA-Seq fastq files were downloaded from the GEO (accession number GSE103097)(Augert, Eastwood et al. 2019). Sequenced reads were aligned to both hg19 and mm10 genomes using STAR. Then the alignment files were processed with bamcmp package(Khandelwal, Girotti et al. 2017) to distinguish between human and mouse reads ("human only" and "human better" reads were retained for further analysis). The featureCounts algorithm(Liao, Smyth et al. 2014) was used to quantify transcript abundance.
To calculate the enrichment of hits from the CRISPR/Cas9 ORY-1001/KDM5-C70 screen, AUCell package(Aibar, Gonzalez-Blas et al. 2017) was utilized. This package has been originally developed for measuring enrichment of given signatures in scRNA-Seq data and it was adapted for our purposes with minor modifications. For all drug-treated CRISPR/Cas9 screens, hits were included if they had a q-value less than 0.25 from the STARS analysis. For the DMSO-treated CRISPR/Cas9 screen, hits were included that had a p-value less than 0.05 as there were very few hits with a q-value of less than 0.25.
To generate heatmaps of relative changes in the SCLC PDX models after ORY-1001 treatment(Augert, Eastwood et al. 2019), the list of genes for each arm of the CRISPR/Cas9 screen was further curated based on the statistical cutoffs listed above. First, genes were ranked in each model and the relative change in ranking was calculated based on the following formula: Relative Change=(Rank Before ORY-1001 Treatment-Rank After ORY-1001 Treatment)/Rank Before ORY-1001 Treatment. Then, the heatmap function in R (version 4.0.2) was used to generate heatmaps and cluster genes/samples based on the relative change.

Single-cell RNAseq analysis of circulating tumor cells (CTC)-derived xenograft (CDX) samples
For the single-cell RNA sequencing data in Figs. 6h,i and Supplementary Figs. 13a-d, the human CDX single-cell RNAseq data was acquired and analyzed as previously reported(Gay, Stewart et al. 2021). Briefly, single-cell count matrixes of SC53-naïve, SC53-cis, SC68-naïve, and sc68-cis were downloaded via GEO Database (GSE138474). Only cells that were analyzed in the original study were kept by matching barcodes. The count matrixes were then transformed and normalized using the "NormalizeData" function with default parameters in the SEURAT v4 R package(Hao, Hao et al. 2021). G2M and S phase gene expression scores were calculated using the "CellCycleScoring" function and subsequently regressed out using "ScaleData" function. Highly variable genes were identified by SEURAT function "FindVariableGenes" with parameter selection.method = "mean.var.plot" and used to perform the Principle component analysis (PCA). The first seven PCs were used for clustering and tSNE transformation using the "RunTSNE" function with default parameters. R software version was 4.0.2.

For manuscripts utilizing custom algorithms or software that are central to the research but not yet described in published literature, software must be made available to editors and reviewers. We strongly encourage code deposition in a community repository (e.g. GitHub). See the Nature Portfolio guidelines for submitting code & software for further information.

Data

Policy information about availability of data

All manuscripts must include a data availability statement. This statement should provide the following information, where applicable:

- Accession codes, unique identifiers, or web links for publicly available datasets
- A description of any restrictions on data availability
- For clinical datasets or third party data, please ensure that the statement adheres to our policy

The CRISPR screening data in Figure 1 was supplied with the submission as Supplementary Table 1. The RNA-seq data in NCI-H1876 sgControl and sgZFP36L1 cells treated with ORY-1001 in Figure 4 are supplied as Supplementary Table 2. The RNA-seq data in CORL47 cells of CRISPR activation of ZFP36L1 (sg1 and sg2) vs. sgControl in Figure 6 are supplied as Supplementary Table 3. All RNA-seq data was deposited to GEO OMNIBUS (Accession Number #GSE202116). There will be no restrictions on data availability.

For Supplementary Fig. 2a, gene expression TPM values of ASCL1, NEUROD1, and POU2F3 were obtained from Broad Institute Cancer Cell Line Encyclopedia (CCLE). For Fig. 3a, RNA-seq data for ZFP36L1 across cancer cell lines was downloaded from CCLE. To identify potential binding sites for LSD1 in target genes (as shown in Supplementary Fig. 3), the Cistrome Data Browser was used. For the RNA expression of ZFP36L1 in human lung tumors in Fig. 3b, the 81 SCLC RNA-Seq data was previously published (George et al. Nature 2015) and publicly available on the cBioPortal. The 747 lung adenocarcinoma samples were from TCGA and publicly available on the cBioPortal. For the correlation analysis of SCLC cell lines in Supplementary Figs. 13e-g, correlations in SCLC cell lines between ZFP36L1 and the AXL, TAP1, or INSM1 were calculated using demap.org. For the correlation analysis of human neuroblastoma tumors in Supplementary Figs. 13h-k, correlations between ZFP36L1 and AXL, TAP1, ASCL1, or INSM1 were calculated using 141 human neuroblastoma from cBioPortal. For the analysis of RNA-Seq data from SCLC PDX models, a previously published RNA-sequencing data set, where SCLC PDX models were treated in vivo and ex vivo with ORY-1001 or the vehicle control, was used (Augert et al. Science Signaling 2019). For the single-cell RNA sequencing data in Figs. 6h,i and Supplementary Figs. 13a-d, the human CDX single-cell RNAseq data was previously published (Gay et al. Cancer Cell 2021) and acquired and analyzed as previously reported.

The RNA-sequencing data generated in this study have been deposited in the GEO database under accession code GSE202116 (https://www.ncbi.nlm.nih.gov/geo/query/acc.cgi?acc=GSE202116). FPKM values and differential expression analyses from the RNA-seq data are included in Supplementary Tables 2 and 3. Log normalized data from the CRISPR screen is included as Supplementary Table 1. Source data for all experiments are included in an excel file. Any other data and materials can be requested from the corresponding author upon reasonable request.

Field-specific reporting

Please select the one below that is the best fit for your research. If you are not sure, read the appropriate sections before making your selection.

☒ Life sciences ☐ Behavioural & social sciences ☐ Ecological, evolutionary & environmental sciences

For a reference copy of the document with all sections, see nature.com/documents/nr-reporting-summary-flat.pdf

Life sciences study design

All studies must disclose on these points even when the disclosure is negative.

Sample size	For large unbiased genome-wide studies including CRISPR/Cas9 genome wide screening and RNA-sequencing experiments, 2 biological replicates (meaning 2 completely independent experiments performed on different days) were performed. For these experiments, sample size was not based on a statistical power calculation but we compared biological replicates and also multiple drug concentrations within each biological replicate for robustness and reproducibility. For all other experiments, sample size was not predetermined and multiple biological replicates were performed (mostly 3, but exact numbers specified in figure legends) and repeated n times based on reproducibility and statistical significance.
Data exclusions	No data were excluded in this study.
Replication	To ensure reproducibility, multiple biological replicates were performed as described above in sample size. All attempts at replication were successful for all experiments in this manuscript.
Randomization	Samples were randomly allocated to experimental groups. For this study, this means that the same cell lines from the same maintenance culture were either infected with sgRNAs or treated with the indicated drugs.
Blinding	For the high-throughput CRISPR screening and RNA-sequencing studies, the experiments were analyzed by Broad Institute (CRISPR screen) or Novogene (RNA-seq) who were blinded to the purpose of the study and the study group. For low throughput experiments, blinding was not possible as the scientist performing the experiments needed to know the conditions to perform the experiment, but the experiments were performed with the appropriate controls, all data are included in this manuscript, and no data were excluded.

Reporting for specific materials, systems and methods

We require information from authors about some types of materials, experimental systems and methods used in many studies. Here, indicate whether each material, system or method listed is relevant to your study. If you are not sure if a list item applies to your research, read the appropriate section before selecting a response.

Materials & experimental systems

n/a	Involved in the study
☐	☒ Antibodies
☐	☒ Eukaryotic cell lines
☒	☐ Palaeontology and archaeology
☒	☐ Animals and other organisms
☒	☐ Human research participants
☒	☐ Clinical data
☒	☐ Dual use research of concern

Methods

n/a	Involved in the study
☒	☐ ChIP-seq
☒	☐ Flow cytometry
☒	☐ MRI-based neuroimaging

Antibodies

Antibodies used	For immunoblot analysis, the following antibodies were used:The primary antibodies used were: rabbit α-ZFP36L1 (Cell Signaling, hBRF1/2, #2119, used at 1:1000), mouse α-INSM1 (SANTA CRUZ, #SC377428, used at 1:1000), rabbit α-SOX2 (Cell Signaling, #3579, used at 1:1000), rabbit α-ASCL1 (Abcam, # ab211327, used at 1:1000), rabbit α-LSD1 (Cell Signaling, #2184), mouse α-Vinculin (Sigma, hVIN-1, # V9131, used at 1:10,000), mouse α-Tubulin (Sigma, B-5-1-2, # T5168, used at 1:5000), mouse α-FLAG (Sigma, clone M2, #F1804, used at 1:2000), rabbit α-V5 (Cell Signaling, #13202, used at 1:1000) and mouse α-b-actin (Sigma, clone AC-15, #A3854, used at 1:25,000). The HRP conjugated secondary antibodies were Goat α-Mouse (Jackson ImmunoResearch 115-035-003) and Goat α-Rabbit (Jackson ImmunoResearch 111-035-003) and were used at 1:5000.<br>For the ChIP-qPCR experiments, anti-KDM1A antibody (Cell signaling, #2139) or a matched isotype control (Cell signaling, #3900) was added to the cells at 1 ug.<br>For the RNA-IP experiments, 2.5 µL (2.5 µg) of mouse anti-FLAG (Sigma, clone M2, #F1804-1MG) antibody or isotype-matched control IgG1 (Cell Signaling #5415S) to the tube for the FLAG IPs, or 5 µL of rabbit anti-V5 (Cell Signaling #13202, used at 1:200) or the isotype-matched IgG control (Cell Signaling #3900) for the V5 IP.
Validation	For the immunoblot analysis, all antibodies (ZFP36L1, ASCL1, SOX2, INSM1, LSD1) have been validated for specificity using either CRISPR knockout or siRNA (most of the validation is actually shown in our paper). The loading controls (actin, vinculin) have been used regularly in many labs. We also knockout validated the LSD1 antibody that was used for ChIP. For the immunocytochemistry (IHC) experiments, ZFP36L1 antibodies were validated using CRISPR knockout isogenic cell pellets in human cell lines and then further validated in mouse cell lines that were known to be ZFP36L1 positive and negative. ASCL1 IHC was validated using known positive and negative ASCL1 human and mouse cell lines. Lastly, we validated that the LSD1 antibody, FLAG antibody, and V5 worked for IP in pilot IP experiments.

Eukaryotic cell lines

Policy information about cell lines

Cell line source(s)	NCI-H1876 (obtained 11/2016), NCI-H1092 (obtained in 11/2018), NCI-H2081 (obtained in 11/2018), NCI-H526 (obtained 10/2019), NCI-H1618 (obtained 1/2020), NCI-H2066 (obtained 1/2020), NCI-H82 (obtained 11/2016), NCI-H446 (obtained 11/2016), and 293FT cells were originally obtained from American Type Culture Collection (ATCC). CORL47 and CORL279 were obtained from Sigma (11/2018). NCI-H69 cells were a kind gift from Dr. Kwok-kin Wong's laboratory (New York University, obtained 8/2014). HEL, THP1 NOMO1, MOL14, K562, HL60 and U937 were a kind gift form Dr. Julie-Aurore Losman's laboratory at DFCI (10/2020). PC9 and HCC4006 (originally from ATCC) cells were a kind gift Dr. Pasi Janne's laboratory at DFCI (7/2020).
Authentication	The main cell lines were from this study were obtained from ATCC or Sigma as described above, which were authenticated by ATCC or Sigma and early passage vials were frozen. All genetic manipulations were done on early passage cell lines and early passage vials of engineered cell lines were frozen. Cells were kept in culture for <4 months (most often <1 month) at which time early passage vials were thawed. Cell lines were not authenticated again after they were initially authenticated by ATCC.
Mycoplasma contamination	Cell lines were tested for mycoplasma and were negative before freezing the early passage vials described above.
Commonly misidentified lines<br>(See ICLAC register)	No commonly misidentified cell lines were used in this study.

March 2021
