# Supplementary material for: Regulation of neuroendocrine plasticity by the RNA-binding protein ZFP36L1
Source: Nat Commun. 2022 Aug 25;13:4998. doi: 10.1038/s41467-022-31998-7 (PMC9411550; doi:10.1038/s41467-022-31998-7)
Supplement: Supplementary file 2 — Peer Review File [file 41467_2022_31998_MOESM2_ESM.pdf]

nature portfolio

Peer Review File

Regulation of Neuroendocrine Plasticity by the RNA-Binding Protein ZFP36L1

Open Access This file is licensed under a Creative Commons Attribution 4.0 International License, which permits use, sharing, adaptation, distribution and reproduction in any medium or format, as long as you give appropriate credit to the original author(s) and the source, provide a link to the Creative Commons license, and indicate if changes were made. In the cases where the authors are anonymous, such as is the case for the reports of anonymous peer reviewers, author attribution should be to 'Anonymous Referee' followed by a clear attribution to the source work. The images or other third party material in this file are included in the article's Creative Commons license, unless indicated otherwise in a credit line to the material. If material is not included in the article's Creative Commons license and your intended use is not permitted by statutory regulation or exceeds the permitted use, you will need to obtain permission directly from the copyright holder. To view a copy of this license, visit http://creativecommons.org/licenses/by/4.0/.

REVIEWER COMMENTS

Reviewer #1 (Remarks to the Author):

The submitted manuscript from Chen et al. looks to identify critical actionable targets which small cell lung cancers (SCLC) are dependent for phenotype maintenance. Currently, SCLC does not have therapeutic solutions, so this paper targets an important area within not only the lung cancer field, but neuroendocrine cancers in general.

Here in, the team investigates a critical chromatin remodeling factor, lysine specific demethylase-1 (LSD-1), known to be important for SCLC survival. They perform a crispr screen in the prescence of LSD-1 +/- KDM5 inhibition to show several genes critical for SCLC models responsiveness to LSD-1 inhibition. Few recently reported genes reported by others including ASCL1 and REST/NOTCH/YAP are identified in the screen - providing regidity to their screen. One gene, and a focus of this manuscript, is ZFP36L1 has not been reported. ZFP36L1 regulates mRNA destabilization via the mRNA 3'UTR and is shown to be signifcantly down regulated in SCLC compared to lung adenocarcinomas. Secondly, the team shows that ZFP36L1 directly regulates these critical genes mentioned above + SOX2 and INSM1 through direct interaction via their 3'UTR. Third, it is shown that up regulation of ZFP36L1 is required for the ant-tumor effects provided by LSD1 inhibition. Finally, ZFP36L1 reactivation/reexpression drives SCLC cells to a non-neuroendocrine 'inflammatory' phenotype. All together, Chen et al. have provided important insight providing rigorous evidence that ZFP36L1 is an important target of LSD1 to maintain a neuroendocrine SCLC phenotype, and up regulation of ZFP36L1 is critical for LSD1 inhibition to induce cell reprograming and anti-tumor action. This work provides a potential novel direction for clinical trial design to test LSD1 inhibitors as mono therapy or in combination to treat SCLC.

This paper is recommended for publication with its current data, though suggest some minor edits that may increase its important to the neuroendocrine field.

1) This paper has denoted the importance of LSD1-ZFP36L1 towards sustained ASCL1 expression and transcriptional function. It is known in SCLC and neuroendocrine cancers that hetergeneity can exist where ASCL1 low neuroendocrine tumors have alternate drivers including NEURD1 transcriptional factors. This is an important discussion point and should be addressed within this manuscript - thoughts on the importance of ZFP36L1/mRNA regulation in NEUROD high ASCL1 low NE tumors?

2) In Fig 4 - adding ASCL1 oe rescue LSD1 inhibition effect would be beneficial to support the current data provided by ZFP36L1 activation/sustained expression.

3) Would the use of LSD1 inhibitors in ASCL1 high SCLC provide combination therapy potential with therapies already approved for NSCLC? This maybe only in the context of EGFRmut NSCLC to SCLC transition tumors????

4) Demonstrating the combination efficacy of LSD1 inhibitors with current IOs (check point inhibitors) would be beneficial. Especially has they are approved for NSCLC.

5) Merge sections for LSD1 inhibition and sensitivity in SCLC/AML as per figure 7 ... A separate AML section takes the focus off SCLC (which is 98% of the paper).

Reviewer #2 (Remarks to the Author):

Chen at all report here that LSD1 regulates the ZFP36L1 as a mechanism of maintaining neuroendocrine differentiation in SCLC. Overall the suggested mechanism is interesting and novel mechanism, but confidence in the conclusions are undermined by significant limitations in the

approaches and concerns over the rigor of the study.

Major Concerns
Throughout the paper, an overwhelming number of data panels are presented with n=2 replicates with sizable error bars. Combined with data presented that does not appear to replicate between panels (for example, significant increase in ZFP36L1 expression in NCI-H1876 cells in Fig 2a was significant but does not appear to replicate in Fig 2d, similar for WWTR1 expression). This raises significant concerns about the rigor of the study and reproducibility of these results.

The sgRNA screen that identified ZFP36L1 as a hit was undertaken in a single SCLC cell line rather than multiple different cell lines. Data presented in Figure 2 across multiple SCLC lines are not especially consistent picture for a correlation between genes dysregulated by LSD1 inhibitors and outcomes of LSD1 inhibitor treatment, with the exception of ASCL1. For example, ZFP36L1 induction by ORY-1001 in panel 2D does not correlate robustly cell count numbers presented in 2C. It seems unlikely that ZFP36L1 expression will be a robust biomarker of response to LSD1 inhibitors.

Small molecule inhibitors are notorious for off target activity. Indeed off target activity of several small molecules account for failure of drugs that demonstrated preclinical efficacy in cell lines to translate to efficacy in clinical trials (Lin A et al, Science Translational Medicine 2019). Crucial experiments linking LSD1 function to ZFP36L need to be performed with CRIPSR knockouts, in multiple SCLC cell lines. For example, LSD1 knockouts presumably grow poorly in culture, does ZFP36L rescue this growth?

Additional Comments:
The authors extensively discuss the results in their screen with REST as a validation that the screen identified relevant components of the pathway at work here. However, the REST one results are significant in only in one of the combination therapy screens, and data for REST are not presented at all in the LSD1 inhibitor monotherapy. As a result, the importance or REST for LSD1 inhibitors is not clearly identified in the screen.

In Figure 3, the authors suggest that ZFP36L1 is repressed in SCLC relative to other cancers. While indeed is expressed at a significantly lower level, whether this represents repression is not clear. The authors should compare ZFP36L1 expression in SCLC compared to normal lung to conclude whether ZFP36L1 is indeed repressed.

Cell lines are used inconsistently throughout the paper. There seems to be a switch in cell lines used, NCI-H1876 and CORL47 are used early in the manuscript and characterized with respect to inhibitors but NCI-H69 appears late in the paper to argue for a ZFP36L-dependent mechanism. Data in all three cell lines should be used throughout the paper to demonstrate reproducibility across cell lines in multiple different experiments.

RIP results for ZFP36L1 for interactions with SOX2 and INSM1 as these were performed without crosslinking in cell lysates, which can lead numerous artifacts (https://pubmed.ncbi.nlm.nih.gov/15388877/). As this is a critical experiment for the proposed mechanism, CLIP data are needed here.

Reviewer #3 (Remarks to the Author):

It has been previously reported that inhibition of LSD1, a histone demethylase, downregulates the transcription factor ASCL1 and neuroendocrine differentiation and cell growth in some small cell lung cancer (SCLC) cell lines and that ASCL1 downregulation correlates with LSD1 inhibitor sensitivity in SCLC cell lines and PDX models. In this study, the authors screen for and uncover critical factors

involved in determining the sensitivity of SCLC to inhibitors of LSD1. The positive selection CRISPR screen identified multiple relevant players that have been implicated in SCLC biology but this study focused on the mRNA-binding protein, ZFP36L1. ZFP36L1 has not previously been identified to play a role in SCLC. The authors show that in at least some SCLC cell line models, ZFP36L1 is a target of LSD1 repression, its expression is induced when LSD1 is inhibited, and it is sufficient when overexpressed to reduce neuroendocrine genes (using ASCL1 as a key marker) and reduce cell growth in SCLC. Knocking down ZFP36L1 when LSD1 is inhibited rescues at least in part some of the effects on ASCL1 and cell growth seen with the LSD1 inhibition. Some mechanistic experiments are performed that show ZFP36L1 binds to SOX2 and INSM1 mRNAs and destabilizes them. ASCL1 mRNA itself is not predicted to be a target but was not explicitly tested. This study adds significant mechanistic insight into LSD1 inhibitor response in subsets of tumors and provides a possible new biomarker for response to the inhibitor. Some evidence is provided that suggests restoring ZFP36L1 expression, as is seen with LSD1 inhibition, induces a non-neuroendocrine phenotype that may improve sensitivity to immune therapies. The study is presented clearly with justification for moving from one experiment to the next. The quality of the experiments is high with important controls included. This work will be received with interest in cancer biology. I have a few suggestions to improve transparency of the findings.

1. Fig. 2B,C: It would be helpful to know what the levels of ASCL1, NEUROD1, and POU2F3 are in the SCLC cell lines used. Currently only relative levels of ASCL1 are shown which does not provide any information on the SCLC subtype. Likewise for Fig 2D-G—what is the variation in ZFP36L1, REST, YAP1, WWTR1 etc across these lines. Do they all express and the levels just go up? Is the expression minimal? Having only the normalized mRNA values do not show this. Possibly a table with RNAseq from their own or public data, or even RTqPCR values not relative to the DMSO treated would increase the information in these graphs (for example use deltaCt not delta deltaCt). This is a suggestion for data throughout the study.

2. For the different SCLC cell lines and PDX used in the study, it is relevant to note what subtype SCLC they are relative to ASCL1 expression at least.

3. In the results section, please state what the 'CRISPR-based SCLC genetically-engineered mouse model" is.

4. It is found that "there are subpopulations of cells within these mouse SCLC tumors where Zfp36l1 is expressed, and these subpopulations have relatively lower expression of Ascl1 (Fig. 3G) suggesting a negative correlation between Zfp36l1 and Ascl1 expression." This is an interesting observation that should be followed up using the extensive SCLC cell line and PDX model RNAseq data to see if it holds true in the human tumors. Also, showing ZFP36L1 expression across the different SCLC subtypes, including the 'inflammatory' type would address the conclusion that "These results demonstrate that restoring ZFP36L1 can functionally induce a non-neuroendocrine state that resembles the "inflammatory" subtype of SCLC." (As stated in the Results page 12.)

5. The supplemental tables where the RNAseq data are presented should be referenced in the results sections and figures where it is used to be more user friendly.

6. Westerns throughout should have MW markers to indicate size.

7. Like stated above for RTqPCR data normalization, Fig 7D,E,G would be better to show normalized values to actin not further normalized to DMSO treatment if possible. It would better reflect what you can see in the WB of some of the cell lines having detectable expression at all. By showing delta delta Ct, this is invisible and it is impossible to see whether cell lines have dramatically different levels of expression.

8. In the AML cell models at the end of the study, I assume they do not work through the ASCL1

mechanism since it is probably not expressed. Is there some other related factor in the AMLs that could serve this role such as ASCL2?

REVIEWER COMMENTS

Reviewer's comments are in plain text and **responses to reviewer's comments are bolded**.

Reviewer #1 (Remarks to the Author):

The submitted manuscript from Chen et al. looks to identify critical actionable targets which small cell lung cancers (SCLC) are dependent for phenotype maintenance. Currently, SCLC does not have therapeutic solutions, so this paper targets an important area within not only the lung cancer field, but neuroendocrine cancers in general.

Here in, the team investigates a critical chromatin remodeling factor, lysine specific demethylase-1 (LSD-1), known to be important for SCLC survival. They perform a crispr screen in the presence of LSD-1 +/- KDM5 inhibition to show several genes critical for SCLC models responsiveness to LSD-1 inhibition. Few recently reported genes reported by others including ASCL1 and REST/NOTCH/YAP are identified in the screen - providing regidity to their screen. One gene, and a focus of this manuscript, is ZFP36L1 has not been reported. ZFP36L1 regulates mRNA destabilization via the mRNA 3'UTR and is shown to be signifcantly down regulated in SCLC compared to lung adenocarcinomas. Secondly, the team shows that ZFP36L1 directly regulates these critical genes mentioned above + SOX2 and INSM1 through direct interaction via their 3'UTR. Third, it is shown that up regulation of ZFP36L1 is required for the ant-tumor effects provided by LSD1 inhibition. Finally, ZFP36L1 reactivation/reexpression drives SCLC cells to a non-neuroendocrine 'inflammatory' phenotype. All together, Chen et al. have provided important insight providing rigorous evidence that ZFP36L1 is an important target of LSD1 to maintain a neuroendocrine SCLC phenotype, and up regulation of ZFP36L1 is critical for LSD1 inhibition to induce cell reprograming and anti-tumor action. This work provides a potential novel direction for clinical trial design to test LSD1 inhibitors as mono therapy or in combination to treat SCLC.

This paper is recommended for publication with its current data, though suggest some minor edits that may increase its important to the neuroendocrine field.

We thank the reviewer for their kind comments.

1) This paper has denoted the importance of LSD1-ZFP36L1 towards sustained ASCL1 expression and transcriptional function. It is known in SCLC and neuroendocrine cancers that heterogeneity can exist where ASCL1 low neuroendocrine tumors have alternate drivers including NEUROD1 transcriptional factors. This is an important discussion point and should be addressed within this manuscript - thoughts on the importance of ZFP36L1/mRNA regulation in NEUROD high ASCL1 low NE tumors?

The reviewer brings up an important point as several studies have now identified different SCLC molecular subtypes each with distinct biology and vulnerabilities, which could lead to opportunities for more tailored targeted therapy approaches. In the original manuscript, we focused specifically on ASCL1-positive SCLCs since LSD1 inhibitor sensitivity was previously more positively correlated with SCLC that express ASCL1(Augert, Eastwood et al. 2019). However, ASCL1 expression itself does not highly correlate with LSD1 inhibitor sensitivity as many ASCL1-positive SCLC models remain inherently resistant to LSD1 inhibitors(Augert, Eastwood et al. 2019), which we confirmed in our study. This observation motivated us to perform our study focusing on ASCL1-positive SCLCs to identify more detailed mechanisms by which LSD1

inhibition blocks neuroendocrine differentiation and causes anti-proliferative/anti-tumor effects.

Based on the reviewer's comment, we tested whether ZFP36L1 is induced in 2 dominantly NEUROD1-positive cell lines (NCI-H446 and NCI-H82) and CORL279 cells that highly express NEUROD1, but also express ASCL1 (see revised Supplementary Fig. 2a for mRNA expression of ASCL1 and NEUROD1 in all cell lines in this study). Relative to ASCL1-positive cell lines (see Fig. 2d), LSD1 inhibition with 2 distinct clinical grade LSD1 inhibitors ORY-1001 and CC-90011 did not induce ZFP36L1 (NCI-H82) or only weakly induced ZFP36L1 (NCI-H446 and CORL279 cells) (compare Supplementary Fig. 5g to Fig. 2d). In contrast, REST expression was more strongly induced by ORY-1001 or CC-90011 in NCI-H446 and CORL279 cells (Supplementary Fig. 5h), which we included as a control to show that LSD1 inhibition was effective at de-repressing canonical LSD1 target genes. Consistent with our cell line data, we also analyzed the RNA-seq data from the SCLC PDX models from Augert et al. Science Signaling 2019(Augert, Eastwood et al. 2019) for SCLC transcription factor subtype expression (ASCL1, NEUROD1, POU2F3), which showed that all SCLC PDX models expressed ASCL1 except for LX33, which expressed NEUROD1 (Supplementary Figs. 14a,b) and did not induce ZFP36L1 upon LSD1 inhibitor treatment (see Fig. 7b and Supplementary Fig. 16a-c). Together these data suggest that LSD1 regulates ZFP36L1 more selectively in ASCL1-positive SCLCs compared to NEUROD1-positive SCLCs.

We have now included the data above in Supplementary Figs. 5g-j and 14a,b of the revised manuscript, which is now described on page 9 of the revised manuscript with the following statement: "Compared with ASCL1-positive SCLC cell lines (Fig. 2d and Supplementary Figs. 5a-c), ZFP36L1 was either not induced or only weakly induced by ORY-1001 or CC-90011 in 3 NEUROD1-positive SCLC cell lines (Supplementary Figs. 5g-j) suggesting that de-repression of ZFP36L1 after LSD1 inhibitor treatment is more selective in ASCL1-positive SCLCs compared to NEUROD1-positive SCLCs." We have also added the following statement on page 16 of the revised discussion: "Our data suggests that ZFP36L1 repression by LSD1 is more selective for the ASCL1-positive SCLCs compared to NEUROD1-positive SCLCs, but future studies using a larger number of NEUROD1-subtype and POU2F3-subtype preclinical models are needed to determine whether LSD1's repression of ZFP36L1 is specific for the ASCL1-subtype."

2) In Fig 4 - adding ASCL1 overexpression rescue LSD1 inhibition effect would be beneficial to support the current data provided by ZFP36L1 activation/sustained expression.

To address this point, we used/developed CORL47 and NCI-H69 cells (both ASCL1-positive cell lines) engineered to inducibly express exogenous ASCL1-FLAG with additional of doxycycline (DOX) under the control of a CMV promoter. We confirmed that addition of DOX successfully induced ASCL1 exogenous expression albeit at lower levels compared to endogenous ASCL1 (see Figure for Reviewer R1a,d). We then performed cell counts in the presence of 2 structurally distinct LSD1 inhibitors in clinical trials (ORY-1001 or CC-90011) with and without DOX to induce exogenous ASCL1 expression. Exogenous ASCL1 expression alone was not sufficient to significantly mitigate the cellular proliferation defects caused by LSD1 inhibition (see Figs. R1b,e). This could be due to technical limitations in that we were not able to sustain expression of exogenous ASCL1 to levels produced in the endogenous context during the course of the experiment (see Figs. R1c,f) thus potentially hindering the ability of exogenous ASCL1 to rescue cellular proliferation. It is possible that it is

biological and that restoring exogenous ASCL1 is not sufficient to restore cellular proliferation in the face of LSD1 inhibition as our data shows that ZFP36L1 regulates mRNA abundance of at least 2 transcription factors necessary for neuroendocrine differentiation (INSM1 and SOX2) and thus exogenous ASCL1 expression may not be sufficient to overcome these effects. However, given the technical limitations described above, we cannot draw this conclusion. Given these technical limitations, we have included these data as a Figure for Review (Fig. R1).

3) Would the use of LSD1 inhibitors in ASCL1 high SCLC provide combination therapy potential with therapies already approved for NSCLC? This maybe only in the context of EGFRmut NSCLC to SCLC transition tumors????

The reviewer brings up a very interesting idea that could apply to *EGFR*-Mutant NSCLC patients with *RB1* and *TP53* loss as these patients have a much higher risk to transdifferentiate to SCLC upon resistance to EGFR TKI(Lee, Lee et al. 2017). We are interested in understanding mechanisms of NSCLC to SCLC trans-differentiation and whether LSD1 inhibition blocks this or alternatively whether loss of genes identified in our screen (e.g. ZFP36L1, REST etc.) can promote this.

Based on the reviewer's suggestion, we have now added the following statements to the revised discussion on page 18: "Moreover, trans-differentiation from *EGFR*-Mutant adenocarcinoma to SCLC occurs as a mechanism of resistance to EGFR inhibitors(Sequist, Waltman et al. 2011, Oser, Niederst et al. 2015) representing plasticity between histological subtypes of lung cancer. Future studies can test whether LSD1 inhibition combined with EGFR inhibitors could prevent trans-differentiation to SCLC in patients with *EGFR*-Mutant adenocarcinoma, which could be particularly beneficial for *EGFR*-Mutant adenocarcinomas with concurrent *RB1* and *TP53* mutations that are at higher risk for SCLC transdifferentiation(Lee, Lee et al. 2017)."

4) Demonstrating the combination efficacy of LSD1 inhibitors with current IOs (check point inhibitors) would be beneficial. Especially has they are approved for NSCLC.

We agree that this is an interesting avenue of research particularly given previous work showing additive to synergistic effects with LSD1 inhibitors and PD-1 checkpoint blockade(Sheng, LaFleur et al. 2018). Our data suggests additional mechanisms by which LSD1 inhibition could contribute to a more highly immunogenic SCLC state given that restoration of ZFP36L1 induces expression of MHC class I genes and that ZFP36L1 expression significant correlates with human SCLCs of the MHC class I high inflammatory subtype (see Fig. 6).

In our revised manuscript, we also analyzed single-cell RNA sequencing data from 2 human SCLC samples before and after cisplatin recurrence [data from Gay et al. Cancer Cell 2021(Gay, Stewart et al. 2021)] for ZFP36L1 expression. We found that ZFP36L1 expression highly correlates with markers of the "inflammatory" phenotype and negatively correlates with ASCL1 and INSM1 in subpopulations of tumor cells that emerge after cisplatin recurrence (see Figs. 6h,i and Supplementary Fig. 13) validating our findings that high ZFP36L1 expression correlates with the "inflammatory" subtype of SCLC at the single-cell level (please also see reviewer 3, point 4 below).

Our manuscript focuses on the identification of the LSD1-ZFP36L1 axis as a cell intrinsic mechanism by which LSD1 inhibition controls proliferation and neuroendocrine differentiation in SCLC and therefore combination efficacy studies with LSD1 inhibitors and IO are beyond the scope of this manuscript.

Based on the reviewer's comment, we have now included a discussion on pages 17,18 of the revised manuscript on the role of how restoring ZFP36L1 through LSD1 inhibition could potentiate activity of IO in SCLC based on the findings above with the following statements: "An "inflammatory" subtype of human SCLC has recently been identified, which has increased immunogenicity, is more responsive to immune checkpoint blockade (ICB), and can emerge in subpopulations of tumors cells that lose ASCL1 after recurrence from chemotherapy(Cai, Liu et al. 2021, Gay, Stewart et al. 2021, Mahadevan, Knelson et al. 2021). We found that ZFP36L1 is highly expressed in the inflammatory subtype of human SCLC and that restoring endogenous ZFP36L1 expression caused loss of neuroendocrine differentiation and promoted an "inflammatory" phenotype. Our results suggest that restoring ZFP36L1 expression in SCLC can drive plasticity toward this "inflammatory" subtype, which could be leveraged therapeutically. LSD1 inhibition is one mechanism by which ZFP36L1 expression can be restored and has been shown to cooperate with PD-1 ICB in preclinical models of melanoma(Sheng, LaFleur et al. 2018). Given our data linking ZFP36L1 to the inflammatory subtype of SCLC which is more responsive to ICB, it is possible that LSD1 inhibition could cooperate with ICB in SCLC."

5) Merge sections for LSD1 inhibition and sensitivity in SCLC/AML as per figure 7 ... A separate AML section takes the focus off SCLC (which is 98% of the paper).

We agree with the reviewer as the AML data was meant to ask whether ZFP36L1 is necessary for LSD1 inhibitor sensitivity in another cancer cell type that is inherently sensitive to LSD1 inhibition. We have now merged these sections (ZFP36L1 induction by LSD1i in SCLC PDX models and ZFP36L1 induction by LSD1i in AML cell lines) in the revised manuscript. We have also cut some of the discussion about AML to focus more on SCLC.

Reviewer #2 (Remarks to the Author):

Chen at all report here that LSD1 regulates the ZFP36L1 as a mechanism of maintaining neuroendocrine differentiation in SCLC. Overall the suggested mechanism is interesting and novel mechanism, but confidence in the conclusions are undermined by significant limitations in the approaches and concerns over the rigor of the study.

Major Concerns
1. Throughout the paper, an overwhelming number of data panels are presented with n=2 replicates with sizable error bars. Combined with data presented that does not appear to replicate between panels (for example, significant increase in ZFP36L1 expression in NCI-H1876 cells in Fig 2a was significant but does not appear to replicate in Fig 2d, similar for WWTR1 expression). This raises significant concerns about the rigor of the study and reproducibility of these results.

We appreciate the reviewer's concerns as our goal is to do science that is rigorous and reproducible. We believe the reviewer is primarily referring to the low-throughput secondary screen of multiple cell lines by RT-qPCR in Figure 2d-g to determine whether hits from our screen are regulated by the LSD1 inhibitor ORY-1001 (or not) across SCLC cell lines. As these experiments were a done as a survey to rule in or rule out genes that were regulated by LSD1 inhibitors across several different SCLC cell lines for further study, we did initially only perform 2 biological replicates for these experiments. We were guided by the results from Fig. 2a (which were performed in 4 biological replicates) where we identified ZFP36L1, REST, and YAP1 as significantly upregulated genes after ORY-1001. Based on the reviewer's comment, we have repeated these experiments in Figs. 2d-g now with either 3 or 4 biological replicates per experiment and the quantitative data is very similar to what we initially observed. Although the reviewer does correctly point out that ZFP36L1 induction in NCI-H1876 cells was significant in Fig. 2a, but not in Fig. 2d of the original manuscript (and conversely for WWTR1 in Fig. 2g vs. Fig. 2a), we respectfully submit that the directionality and magnitude of induction of target genes were very similar for all experiments demonstrating the reproducibility of the findings between experiments. With the additional biological replicate/s, changes in ZFP36L1 and WWTR1 are now both statistically significant in revised Figs. 2d,g for the cell lines indicated. These data are now included in the revised manuscript in Figs. 2d-g.

For the ChIP-qPCR experiments in Figs. 2h,i of the original and revised manuscript, the LSD1 ChIP-qPCR experiments were initially performed in 4 biological replicates for NCI-H1876 cells (Fig. 2h) and in 4 biological replicates for CORL47 cells (Fig. 2i). For the CORL47 and NCI-H1876 cells control IgG ChIP-qPCR experiments, only 2 biological replicates were performed. We apologize that this was incorrectly simplified in the figure legend originally stating that 2 biological replicates were performed (as this was the minimum performed for each condition). We apologize for this oversight and now have corrected this in the revised manuscript figure legends 2h,i.

Our genome-wide CRISPR/Cas9 screen (Fig. 1) and RNA-sequencing experiments (Figs. 4&6) were performed in 2 biological replicates. However, each experiment was performed with either multiple different drug concentrations or with 2 independent sgRNAs and the results were similar irrespective of the drug concentration or sgRNA used demonstrating robustness of the phenotypes irrespective of the drug concentration or sgRNA used. Specifically, each biological replicate of the genome-

wide CRISPR/Cas9 sgRNA screen (Fig. 1 and Supplementary Fig. 1) was performed splitting into 4 independent pools of cells that were treated with 2 low concentrations of ORY-1001 +/- KDM5-C70. The hits in the ORY-1001 monotherapy arms were very similar to one another and the hits in the combination arms were very similar to one another. Similarly, for RNA-seq with CORL47 ZFP36L1 CRISPR activated cells (Fig. 6), we observed similar phenotypes with respect to neuroendocrine differentiation and immunogenicity irrespective of the sgRNA used. Lastly, for the RNA-seq experiment in Fig. 4 with the NCI-H1876 ZFP36L1 isogenic cells treated with ORY-1001, we observed similar phenotypes with ORY-1001 at 1 nM or 100 nM. We had not included the data with 100 nM ORY-1001 in the original manuscript, but have now included the data with ORY-1001 100 nM in the revised manuscript in Supplementary Figs. 9 and 10 and Supplementary Table 2. The results are similar to the results with ORY-1001 1 nM again demonstrating robustness of the findings irrespective of the drug concentration used.

2. The sgRNA screen that identified ZFP36L1 as a hit was undertaken in a single SCLC cell line rather than multiple different cell lines.

The reviewer brings up a good point as a hit from a CRISPR/Cas9 screen that only validates in the screening cell line (and not other cell lines in that given cancer type) is likely not robust and meaningful. This was not the case for ZFP36L1 as we broadly validated this finding functionally in several human SCLC cell lines. Furthermore, we used several other models throughout our manuscript to correlate our functional data with SCLC cell lines, mouse models of SCLC, human TMAs of SCLC and lung adenocarcinoma, human RNA-seq data of SCLC and adenocarcinoma, SCLC PDX models, human single-cell RNA-sequencing after cisplatin recurrence, INSM1-positive SCLC cell line models, and neuroblastoma human tumors. We even extended our findings to show that ZFP36L1 is necessary for LSD1 inhibitor sensitivity in AML, which like SCLC, is inherently highly sensitive to LSD1 inhibitors.

We chose to initially perform the LSD1 inhibitor resistance screen in NCI-H1876 cells as NCI-H1876 cells are an ASCL1-positive SCLC cell line that is highly sensitive to LSD1 inhibitors [sensitive at very low nanomolar concentrations where nearly all other cancer cell lines apart from some SCLCs and AMLs are inherently resistant(Augert, Eastwood et al. 2019)], and are highly efficient at CRISPR/Cas9 editing and therefore are amenable to CRISPR/Cas9 high-throughput screening. These 3 criteria (LSD1 inhibitor sensitive, ASCL1-positive, and efficient CRISPR/Cas9 editing capacity) are actually challenging criteria to fulfill as ASCL1-positive SCLC cell lines [the SCLC lines that are more highly correlated with sensitivity to LSD1 inhibitors(Augert, Eastwood et al. 2019)], are notoriously inefficient at CRISPR/Cas9 editing(Huang, Klingbeil et al. 2018). Therefore we decided to perform the screen in NCI-H1876 cells at 2 different low nanomolar concentrations of ORY-1001 with and without KDM5-C70 each in 2 biological replicates. This amounts to 8 genome-wide resistance screens in total with each genome-wide screen carrying a different pool of cells infected with the whole genome sgRNA library. Our goal was to find hits (genes) that when inactivated conferred ORY-1001 resistance at both drug concentrations (with or without KDM5 inhibitors) with the ultimate goal being to validate findings across several models (see above). As shown in Fig. 1, ZFP36L1 scored as a top hit across all arms of the screen fulfilling this criteria, which we then validated functionally in several human SCLC cell lines. Therefore, although our discovery of ZFP36L1 as a gene necessary for LSD1 inhibitor sensitivity in SCLC was initially from a single SCLC cell line, our data

supports a broad role for LSD1-ZFP36L1 in regulating neuroendocrine differentiation and proliferation in ASCL1-positive SCLCs.

3. Data presented in Figure 2 across multiple SCLC lines are not especially consistent picture for a correlation between genes dysregulated by LSD1 inhibitors and outcomes of LSD1 inhibitor treatment, with the exception of ASCL1. For example, ZFP36L1 induction by ORY-1001 in panel 2D does not correlate robustly cell count numbers presented in 2C. It seems unlikely that ZFP36L1 expression will be a robust biomarker of response to LSD1 inhibitors.

The reviewer's statement is correct that only downregulation of ASCL1 after LSD1 inhibitor treatment highly correlates with LSD1 inhibitor sensitivity in the cell lines in Fig. 2 as we stated in the original manuscript: "Consistent with previous findings(Augert, Eastwood et al. 2019), ORY-1001 repression of ASCL1 correlated with ORY-1001 sensitivity."

We apologize for any misconception of what the ZFP36L1 expression data after ORY-1001 treatment showed in Fig. 2. We agree with the reviewer that the ZFP36L1 data in Fig. 2d does not highly correlate with ORY-1001 sensitivity in Fig. 2c and we did not claim that it did in the results section describing Fig. 2. Later in the manuscript, we were curious to further investigate the relationship between LSD1 inhibitor sensitivity and ZFP36L1 induction as two of the three cell lines with the highest magnitude of ZFP36L1 induction after ORY-1001 in Fig. 2d (CORL47 and NCI-H2081) were relatively sensitive to ORY-1001 (see Fig. 2c), which is why we chose to further ask unbiasedly whether a correlation existed between ZFP36L1 induction and ORY-1001 sensitivity using RNA-seq data from SCLC PDX models treated with ORY-1001(Augert, Eastwood et al. 2019) in Fig. 7. Taking all of the hits with statistical significance (q-value<0.25) from our screen, we asked which "hits" were transcriptionally induced by LSD1 inhibitors in these models and then correlated this with ORY-1001 sensitivity in each PDX model. This analysis revealed that ZFP36L1 and REST induction were most robustly induced by ORY-1001 across all models as ZFP36L1 and REST both clustered all the way to the right (highest upregulation) in the unsupervised hierarchical clustering analysis (see fig. 7b and S16a-c). Interestingly, we found that the one model that is completely resistant to LSD1 inhibitors (LX33), showed induction of REST, but no change in ZFP36L1. Our findings in AML cell lines also support a correlation between ZFP36L1 induction and LSD1 inhibitor sensitivity.

We appreciate the reviewer's comment as we don't want readers to overinterpret our findings. Based on the reviewer's comment, we have now omitted the statement "We observed a positive correlation between ZFP36L1 induction and inhibition of cellular proliferation after LSD1 inhibitor treatment" on page 12 of the original manuscript, which was originally a topic sentence intended to introduce our studies exploring relationships in SCLC PDX models. The introduction to that paragraph now reads on page 13, "To further investigate whether ZFP36L1 is induced by LSD1 inhibitors in SCLC preclinical models *in vivo*, we interrogated a publicly available RNA-seq dataset of SCLC patient-derived xenograft (PDX) models that were treated with ORY-1001 and displayed a variable response to ORY-1001(Augert, Eastwood et al. 2019)."

We have also changed the concluding sentence of that section on page 14 of the revised manuscript. Originally it read "Together, these data suggest that ZFP36L1 induction after ORY-1001 treatment is a candidate biomarker to predict response to

ORY-1001." and now reads "Together, these data show that ZFP36L1 induction after ORY-1001 treatment correlates with response to ORY-1001 in SCLC PDX models."

We have also softened the following statement in our discussion (page 19) to exclude "cell lines" from the statement in our revised manuscript: Original manuscript statement: "We found that ZFP36L1 induction after LSD1 inhibitor treatment highly correlated with response in SCLC PDX models and SCLC cell lines" now reads as "We found that ZFP36L1 induction after LSD1 inhibitor treatment correlated with response in SCLC PDX models."

We also discuss in the original and revised manuscript that more data from SCLC patients (ideally from patient's tumors on LSD1 inhibitor clinical trials before and after treatment with LSD1 inhibitors) are needed to determine whether ZFP36L1 induction could be a useful biomarker of response in SCLCs to LSD1 inhibitors. This is on page 19 of the original and revised manuscript with the following statements: "We were unable to find a single biomarker that was predictive of response. Our data confirms previous findings that ASCL1 down-regulation after LSD1 inhibitor treatment correlates with sensitivity(Augert, Eastwood et al. 2019), but ASCL1 expression alone was not predictive of LSD1 inhibitor sensitivity. We found that ZFP36L1 induction after LSD1 inhibitor treatment highly correlated with response in SCLC PDX models. Future studies are needed to identify predictive biomarkers to identify subsets of SCLC patients that are more likely to have benefit from LSD1 inhibitors."

We hope these changes help clarify the interpretation of our data and again apologize if our writing led to any misconceptions in the original manuscript.

4. Small molecule inhibitors are notorious for off target activity. Indeed off target activity of several small molecules account for failure of drugs that demonstrated preclinical efficacy in cell lines to translate to efficacy in clinical trials (Lin A et al, Science Translational Medicine 2019). Crucial experiments linking LSD1 function to ZFP36L need to be performed with CRIPSR knockouts, in multiple SCLC cell lines. For example, LSD1 knockouts presumably grow poorly in culture, does ZFP36L rescue this growth?

In our original manuscript, we chose to primarily focus on 2 structurally distinct and highly selectively LSD1 inhibitors, ORY-1001 and CC-90011, that are in clinical trials for SCLC patients. Previous studies have shown that some SCLC preclinical models are highly sensitive to each of these inhibitors. Hence, given the clinical relevance of LSD1 inhibitors in SCLC, we wanted to gain mechanistic insights on: 1. Why some SCLCs are highly sensitive to LSD1 inhibitors; and 2. How LSD1 inhibitors block proliferation and neuroendocrine differentiation. Previous data does show that each of these inhibitors are highly selective for LSD1(Maes, Mascaro et al. 2018, Augert, Eastwood et al. 2019, Kanouni, Severin et al. 2020).

We agree with the reviewer that small molecule inhibitors can have off-target effects and that the LSD1 CRISPR/Cas9 knockout experiments are important. To address the reviewer's comment, we first used NCI-H526 cells, a POU2F3-positive SCLC cell line that is highly efficient at CRISPR/Cas9 editing(Huang, Klingbeil et al. 2018), to find sgRNAs that effectively inactivated LSD1. We then took the most highly effective LSD1 sgRNA (sg4) and transduced lentiviruses encoding LSD1 sg4 or a non-targeting sgRNA control into the ZFP36L1 CRISPR knockout CORL47 and NCI-H1876 isogenic cell lines previously used in our study (see Fig. 4c and Supplementary Fig. 6a for ZFP36L1

isogenic cell lines). The results show that ZFP36L1 inactivation significantly mitigated the anti-proliferative effects of LSD1 CRISPR inactivation in NCI-H1876 cells and ZFP36L1 CRISPR inactivation nearly significantly (p=0.055) mitigated the anti-proliferative effect of LSD1 knockout in CORL47 cells. Similar to our findings with the LSD1 inhibitors (ORY-1001 and CC-90011), LSD1 CRISPR inactivation significantly induced ZFP36L1 mRNA expression in both CORL47 and NCI-H1876 cells.

Achieving effective CRISPR inactivation of LSD1 in these cell lines that were previously CRISPR inactivated for ZFP36L1 was incredibly technically challenging and yielded low knockout efficiency, particularly in NCI-H1876 cells which likely explains the very modest anti-proliferative effects and ZFP36L1 mRNA induction in the cells infected with the LSD1 sgRNA. This could be due to the modest CRISPR/Cas9 activity in NCI-H1876 cells at baseline and that prior CRISPR/Cas9 editing of ZFP36L1 in these cells limited the ability to edit an additional gene. Lastly, it is possible that LSD1 knockout is not tolerated and therefore cells quickly select against cells that successfully knockout LSD1. Nonetheless, these experiments support our data with LSD1 small molecule inhibitors by showing that ZFP36L1 is important for the anti-proliferative effects of LSD1 inactivation and that LSD1 inactivation induces ZFP36L1 mRNA expression.

These new data are now included in Supplementary Fig. 8 of the revised manuscript and described on pages 9 and 10 of the revised manuscript with the following statement: "Similar to the LSD1 inhibitor phenotypes above, CRISPR inactivation of ZFP36L1 significantly attenuated the anti-proliferative effects caused by LSD1 CRISPR inactivation in NCI-H1876 cells and nearly significantly attenuated the proliferation defects caused by LSD1 inactivation in CORL47 cells (p=0.055) (Supplementary Figs. 8b-d,f-g). LSD1 CRISPR inactivation also induced ZFP36L1 expression in CORL47 and NCI-H1876 cells (Supplementary Figs. 8e,h)."

Additional Comments:
5. The authors extensively discuss the results in their screen with REST as a validation that the screen identified relevant components of the pathway at work here. However, the REST one results are significant in only in one of the combination therapy screens, and data for REST are not presented at all in the LSD1 inhibitor monotherapy. As a result, the importance or REST for LSD1 inhibitors is not clearly identified in the screen.

We apologize if our discussion of previous work in the field related to REST led to any misconceptions about the findings from our screen. REST scored as a statistically significant hit (q-value<0.25, see method for statistical cut-off in STARS analysis used to call hits) in both arms of the combination screen (see Supplementary Figs. 1g,h and Supplementary Table 1), which we were encouraged by and stated accurately in the original manuscript with the following statement which is now on page 6 of the revised manuscript: "A top hit in the combined treatment arm was REST (Supplementary Figs. 1g,h), a transcriptional repressor of neuronal genes(Schoenherr and Anderson 1995). REST is an LSD1-target gene(Augert, Eastwood et al. 2019, Jin, Ma et al. 2019) and a NOTCH-target gene that is necessary and sufficient to induce a non-neuroendocrine subpopulation of cells during SCLC tumorigenesis(Lim, Ibaseta et al. 2017, Jin, Ma et al. 2019). These results are consistent with previous data demonstrating that LSD1 and KDM5 inhibitors mediate their anti-proliferative effects through a NOTCH-dependent mechanism(Augert, Eastwood et al. 2019, Oser, Sabet et al. 2019) and suggest that the NOTCH-dependent mechanism converges on REST."

The data on REST in the ORY-1001 monotherapy arms was included in Supplementary Table 1 in the original manuscript. REST was enriched in the ORY-1001 monotherapy arms (scored at 1428 in 1 nM ORY-1001 and 135 in 100 nM 1 nM ORY-1001 out of roughly 19,000 genes targeted by the sgRNA library-see Supplementary Table 1 of the original and revised manuscript), but was not a statistically significant hit and we never stated that it was. We actually found it interesting that REST more selectively scored as a statistically significant hit only in the combination arm as we previously found that combined LSD1 and KDM5 inhibition completely blocked neuroendocrine differentiation while either drug alone had only partial effects (Oser et al. Genes and Development 2019, Figure S4). This potentially suggested that genes that scored in the combination arm were more closely linked to genes that oppose neuroendocrine differentiation, such as was seen for REST as described above.

Later in the manuscript, we do mention REST as a positive control for RT-qPCR experiments with LSD1 inhibitors as REST was previously identified as an LSD1 target gene(Jin, Ma et al. 2019), but we did not state that REST was functionally required for resistance with LSD1 inhibitor monotherapy. We apologize if there was any confusion on these points addressed above.

6. In Figure 3, the authors suggest that ZFP36L1 is repressed in SCLC relative to other cancers. While indeed is expressed at a significantly lower level, whether this represents repression is not clear. The authors should compare ZFP36L1 expression in SCLC compared to normal lung to conclude whether ZFP36L1 is indeed repressed.

The reviewer brings up a good point. We did find strong ZFP36L1 staining in the bronchial lining in normal mouse lung bronchial epithelial cells present in our original IHC sections with relatively repressed ZFP36L1 staining in the adjacent SCLC tumors. In fact, after optimizing the ZFP36L1 IHC stain using CRISPR/Cas9 ZFP36L1 knockout isogenic PC9 cell blocks, this bronchial staining pattern encouraged us that our IHC on tissue specimens was working as we expected ZFP36L1 staining in normal lung given public databases such as GTEX portal showing that ZFP36L1 should be expressed in the normal lung. We have now included this IHC data in Supplementary Fig. 4a of the revised manuscript. In response to the reviewer's comment, we did additional ZFP36L1 IHC on human SCLC lung tumors that included normal lung and again found strong bronchial staining for ZFP36L1 and relatively repressed staining of ZFP36L1 in the tumor. Representative images from these experiments are now shown in Supplementary Fig. 4b and described on pages 8 of the revised manuscript with the following statement: "In contrast, ZFP36L1 protein is highly expressed in normal bronchial epithelial cells in both mouse and human lungs (Supplementary Figs. 4a,b) suggesting that ZFP36L1 protein is repressed in SCLC."

7. Cell lines are used inconsistently throughout the paper. There seems to be a switch in cell lines used, NCI-H1876 and CORL47 are used early in the manuscript and characterized with respect to inhibitors but NCI-H69 appears late in the paper to argue for a ZFP36L-dependent mechanism. Data in all three cell lines should be used throughout the paper to demonstrate reproducibility across cell lines in multiple different experiments.

The reviewer points out that we started using NCI-H69 cells later in the manuscript when testing the effects of re-introducing exogenous ZFP36L1 into SCLC cell lines. We initially did attempt these experiments in NCI-H1876 and CORL47 (the 2 workhorse cell lines that we used throughout the manuscript). We were able to generate NCI-H1876

cells that inducibly expressed DOX-ON ZFP36L1 WT or the ZFP36L1 mRNA-binding mutant and the proliferation data is consistent with the data in CORL47 and NCI-H69 showing that exogenous expression of ZFP36L1 WT, but not the mRNA-binding mutant, significantly inhibited proliferation (see Fig. R2a-c for reviewer below). However, there was a technical caveat to this experiment (that we were unable to overcome for unclear reasons) in that protein levels of ZFP36L1 were significantly lower for the mRNA-binding mutant compared WT (Fig. R2b), which confounds the interpretation of the proliferation experiments above as we were concerned that the inability of the mRNA-binding mutant to rescue could be due to the much lower protein expression of the mutant compared to the WT. We also tried constitutive expression with the plx304 vector of ZFP36L1 (WT and mutant) in NCI-H1876 cells and were never able get ZFP36L1 WT cells after antibiotic selection possibly because of the tumor suppressive effects of ZFP36L1 WT in NCI-H1876 cells. For these reasons, we then engineered isogenic ZFP36L1 NCI-H69 cells and used these cells along with CORL47 isogenic cells to study the effects of restoring ZFP36L1 expression.

We did not use NCI-H69 cells earlier in the paper to do CRISPR experiments as NCI-H69 are highly inefficient at CRISPR editing and therefore it is not possible to effectively knockout targets with sgRNAs in these cells. Please see Fig. R3a below showing a quantitative GFP reporter assay for Cas9 activity where NCI-H69 Cas9 cells only effectively edit 14% of cells over 34 days, which is incompatible with using them for CRISPR knockout experiments. NCI-H82 cells, which are highly efficient at CRISPR editing, are included as a positive control (Fig. R3b).

In response to the reviewer's comment, we performed additional SOX2 and INSM1 siRNA experiments in NCI-H69 cells and the results are consistent with the NCI-H1876 and CORL47 cells showing decreased ASCL1 protein levels after knockdown of SOX2 or INSM1. These data are included in Figs. 5n,o of the revised manuscript.

In summary, when technically possible we tried our best to stay consistent with the cell lines used throughout the manuscript and always used at least 2 different SCLC cell lines to perform the isogenic functional experiments.

8. RIP results for ZFP36L1 for interactions with SOX2 and INSM1 as these were performed without crosslinking in cell lysates, which can lead numerous artifacts (https://pubmed.ncbi.nlm.nih.gov/15388877/). As this is a critical experiment for the proposed mechanism, CLIP data are needed here.

The reviewer is correct that we did not perform our RIP experiments with crosslinking. However, to control for non-specific interactions, we performed these experiments using both ZFP36L1-WT and a ZFP36L1 mRNA-binding mutant that contained point mutations that specifically disrupt ZFP36L1's ability to bind to 3' UTRs(Loh, Sun et al. 2020). We also performed these experiments using both FLAG and V5 as epitopes with known positive (e.g. E2F1, CDK2) and negative controls and found that only ZFP36L1 WT, but not the ZFP36L1 mRNA-binding mutant bound to SOX2 and INSM1. As an additional negative control, during the revisions we used these V5-tagged samples to test ASCL1, which does not contain canonical ZFP36L1 binding site in its 3' UTR and found that ASCL1 mRNA was not pulled down by either ZFP36L1 WT nor the mRNA-binding mutant (see Supplementary Fig. 12c in the revised manuscript). Importantly, in the original manuscript we also used an orthogonal approach again with both ZFP36L1 WT and the mRNA-binding mutant to ask whether ZFP36L1 functionally destabilizes the

3’UTRs of SOX2 and INSM1 *in vivo* (in cells). To do this, we performed the luciferase reporter assay in 293T cells shown in Figs. 5l,m. In these experiments, we cloned the 3’ UTRs of SOX2 or INSM1 into a luciferase fusion construct, expressed these 3’ UTRs fused to luciferase in 293T cells that were stably engineered to express ZFP36L1 WT or the ZFP36L1 mRNA-binding mutant. We found 293T cells expressing ZFP36L1 WT, but not the ZFP36L1 mRNA-binding mutant, could destabilize the 3’UTR of SOX2 or INSM1. Together with our RIP data showing binding of ZFP36L1 WT, but not the ZFP36L1 mRNA-binding mutant, to SOX2 and INSM1, we provide 2 orthogonal lines of evidence (biochemically from CORL47 cells and a functional *in vivo* assay using the 3’ UTRs of SOX2 and INSM1 in 293T cells) to support a functional interaction between ZFP36L1 and the 3’ UTRs of SOX2 and INSM1.

Reviewer #3 (Remarks to the Author):

It has been previously reported that inhibition of LSD1, a histone demethylase, downregulates the transcription factor ASCL1 and neuroendocrine differentiation and cell growth in some small cell lung cancer (SCLC) cell lines and that ASCL1 downregulation correlates with LSD1 inhibitor sensitivity in SCLC cell lines and PDX models. In this study, the authors screen for and uncover critical factors involved in determining the sensitivity of SCLC to inhibitors of LSD1. The positive selection CRISPR screen identified multiple relevant players that have been implicated in SCLC biology but this study focused on the mRNA-binding protein, ZFP36L1. ZFP36L1 has not previously been identified to play a role in SCLC. The authors show that in at least some SCLC cell line models, ZFP36L1 is a target of LSD1 repression, its expression is induced when LSD1 is inhibited, and it is sufficient when overexpressed to reduce neuroendocrine genes (using ASCL1 as a key marker) and reduce cell growth in SCLC. Knocking down ZFP36L1 when LSD1 is inhibited rescues at least in part some of the effects on ASCL1 and cell growth seen with the LSD1 inhibition. Some mechanistic experiments are performed that show ZFP36L1 binds to SOX2 and INSM1 mRNAs and destabilizes them. ASCL1 mRNA itself is not predicted to be a target but was not explicitly tested. This study adds significant mechanistic insight into LSD1 inhibitor response in subsets of tumors and provides a possible new biomarker for response to the inhibitor. Some evidence is provided that suggests restoring ZFP36L1 expression, as is seen with LSD1 inhibition, induces a non-neuroendocrine phenotype that may improve sensitivity to immune therapies. The study is presented clearly with justification for moving from one experiment to the next. The quality of the experiments is high with important controls included. This work will be received with interest in cancer biology. I have a few suggestions to improve transparency of the findings.

We thank the reviewer for their kind comments. Based on the comment above about ASCL1 mRNA, we did formally test whether ASCL1 mRNA was pulled down with ZFP36L1 in our RNA-IP experiments. Consistent with the absence of canonical ZFP36L1 binding sites in the 3' UTR of ASCL1, we did not observe ASCL1 mRNA pulled down with ZFP36L1. This data is shown in Supplementary Fig. 12c in the revised manuscript with the following statement on page 11: "To test this, we performed RNA-binding protein immunoprecipitation (RIP) with FLAG-tagged or V5-tagged ZFP36L1 WT or the ZFP36L1 mRNA-binding mutant in CORL47 cells followed by RT-qPCR for SOX2 and INSM1. RT-qPCR was also performed for previously identified ZFP36L1 targets including E2F1 and CDK2(Loh, Sun et al. 2020) as positive controls and ASCL1 and GAPDH as negative controls. ZFP36L1 WT, but not the ZFP36L1-mRNA binding mutant, bound to the mRNAs of SOX2, INSM1, E2F1, and CDK2, but not ASCL1 or GAPDH (Figs. 5i,j and Supplementary Figs. 12b,c)."

1. Fig. 2B,C: It would be helpful to know what the levels of ASCL1, NEUROD1, and POU2F3 are in the SCLC cell lines used. Currently only relative levels of ASCL1 are shown which does not provide any information on the SCLC subtype. Likewise for Fig 2D-G—what is the variation in ZFP36L1, REST, YAP1, WWTR1 etc across these lines. Do they all express and the levels just go up? Is the expression minimal? Having only the normalized mRNA values do not show this. Possibly a table with RNAseq from their own or public data, or even RTqPCR values not relative to the DMSO treated would increase the information in these graphs (for example use deltaCt not delta deltaCt). This is a suggestion for data throughout the study.

We thank the reviewer for their suggestion and agree that this is helpful for understanding baselines for SCLC transcription factor subtype expression and

baseline expression of genes necessary for response to LSD1 inhibitors including *ZFP36L1*, *REST*, *YAP1*, and *WWTR1*. We have now used RNA-seq data from CCLE and show mRNA expression of SCLC subtype transcription factors ASCL1, NEUROD1, and POU2F3 of the cell lines used in this study (see Supplementary Fig. 2a). We have also now included ΔC_T method followed by $2^{-\Delta CT}$ (to understand relative levels of the gene of interest compared to *ACTB*) data for all RT-qPCR experiments in this study of LSD1 target genes (ZFP36L1, REST, YAP1, and WWTR1) in the supplemental figures (Supplementary Figs. 2b-f, 5i,j). Overall, ZFP36L1, REST, YAP1, and WWTR1 are highly repressed in all ASCL1-positive cell lines used in this study and LSD1 inhibition causes expression to go up.

2. For the different SCLC cell lines and PDX used in the study, it is relevant to note what subtype SCLC they are relative to ASCL1 expression at least.

Please also see response to comment 1 above. We have now included expression of the transcription factor subtypes for all cell lines used in this study (Supplementary Fig. S2a). We have also now included transcription factor subtype expression of the SCLC PDX models in Fig. 7 in Supplementary Figs. 14a,b.

3. In the results section, please state what the ‘CRISPR-based SCLC genetically-engineered mouse model” is.

We apologize for this. Our laboratory developed a SCLC genetically-engineered mouse model using CRISPR/Cas9 where an adenovirus encoding sgRNAs targeting *Rb1*, *Trp53*, and *Rbl2* and Cre-recombinase is intratracheally injected into lox-stop-lox Cas9 mice, which results in autochthonous lung tumor formation and spontaneously metastasizes to the liver(Oser, Sabet et al. 2019). We have now included this description in the results section of the revised manuscript on page 8 with the following statement: “Moreover, ZFP36L1 protein was largely absent in the majority of cells in mouse SCLC lung tumors derived from a CRISPR-based SCLC genetically-engineered mouse model (Figs. 3e,f) where autochthonous lung tumors are generated by CRISPR/Cas9 editing of *Rb1*, *Trp53*, and *Rbl2* in somatic cells in the lung(Oser, Sabet et al. 2019, Hong, Knelson et al. 2021).”

4. It is found that “there are subpopulations of cells within these mouse SCLC tumors where Zfp36l1 is expressed, and these subpopulations have relatively lower expression of Ascl1 (Fig. 3G) suggesting a negative correlation between Zfp36l1 and Ascl1 expression.” This is an interesting observation that should be followed up using the extensive SCLC cell line and PDX model RNAseq data to see if it holds true in the human tumors. Also, showing ZFP36L1 expression across the different SCLC subtypes, including the ‘inflammatory’ type would address the conclusion that “These results demonstrate that restoring ZFP36L1 can functionally induce a non-neuroendocrine state that resembles the “inflammatory” subtype of SCLC.” (As stated in the Results page 12.)

We thank the reviewer for this suggestion. In our original manuscript, we had analyzed RNA-seq data from human SCLC that were characterized as either MHC class I high (which corresponds to the inflammatory subtype of SCLC) or MHC class I low by IHC and found that ZFP36L1 expression was significantly increased in MHC class I high compared to MHC class I low human SCLCs. This is shown in Fig. 6g of the original and revised manuscript and supports our conclusion that ZFP36L1 expression is correlated

with the "inflammatory" SCLC subtype. To further explore whether ZFP36L1 expression is correlated with the inflammatory subtype of SCLC, we now used single-cell RNA sequencing data on 2 human SCLC CDX tumors prior to treatment with chemotherapy and after chemotherapy recurrence as a previous study found that an ASCL1 low population, inflammatory expression high, subpopulation of cells emerged after recurrence from chemotherapy(Gay, Stewart et al. 2021). We hypothesized that this subpopulation would highly express ZFP36L1. We found that ZFP36L1 was selectively expressed in this subpopulation of tumor cells after chemotherapy recurrence that had high expression of inflammatory markers (HLA genes) and low expression of ASCL1 and INSM1 thereby validating our findings from mouse tumors in human samples and showing correlations of ZFP36L1 expression with the inflammatory subtype in human SCLC tumors. This data is shown in Fig. 6h,i and Supplementary Fig. 13a-d of the revised manuscript with the following statement on page 13 of the revised manuscript: "We then analyzed single-cell RNA-seq data from 2 paired human SCLCs before and after cisplatin recurrence where a subpopulation of ASCL1/INSM1-low, inflammatory-high tumor cells emerged after cisplatin recurrence(Gay, Stewart et al. 2021). Consistent with our data above, ZFP36L1 was expressed in the inflammatory tumor cell subpopulation that emerged after cisplatin recurrence while its expression was largely absent in the ASCL1/INSM1-high neuroendocrine populations (Figs. 6h,i and Supplementary Figs. 13a-d)."

Consistent with data above, RNA-seq data from SCLC cell lines (from depmap.org) showed that ZFP36L1 expression positively correlated with AXL and TAP1 (two markers of the inflammatory subtype of SCLC) and negatively correlated with INSM1. ZFP36L1 expression did negatively correlate with ASCL1 in all cell lines with statistical significance and also negatively correlated with ASCL1 in SCLC cell lines, but its negative correlation with ASCL1 in SCLC did not reach statistical significance. This data is shown is Supplementary Figs. 13e-g of the revised manuscript with the following statement on page 13 of the revised manuscript: "Lastly, ZFP36L1 expression significantly positively correlated with markers of the inflammatory subtype including AXL and TAP1(Gay, Stewart et al. 2021, Mahadevan, Knelson et al. 2021) and significantly negatively correlated with INSM1 in SCLC cell lines (Supplementary Figs. 13e-g)."

We also analyzed publicly available RNA-seq data from human neuroblastomas (another neuroendocrine tumor) and found significant negative correlations of ASCL1 or INSM1 with ZFP36L1 and positive correlations of AXL or TAP1 with ZFP36L1 supporting this correlative relationship in other neuroendocrine tumors. This data is shown in Supplementary Fig. 13h-k of the revised manuscript with the following statement on page 13: "These correlations also held true in human neuroblastoma; another neuroendocrine tumor (Supplementary Figs. 13h-k)."

These results are summarized on page 13 of the revised manuscript as follows: "Together, these results demonstrate that restoring ZFP36L1 can functionally induce a non-neuroendocrine state that resembles the "inflammatory" subtype of SCLC and ZFP36L1 expression is highly correlated with the "inflammatory" subtype of SCLC in human tumors."

5. The supplemental tables where the RNAseq data are presented should be referenced in the results sections and figures where it is used to be more user friendly.

We apologize for this oversight and have now referenced the Supplementary Tables in the results section.

6. Westerns throughout should have MW markers to indicate size.

We have added molecular weights to the immunoblots throughout the figures.

7. Like stated above for RTqPCR data normalization, Fig 7D,E,G would be better to show normalized values to actin not further normalized to DMSO treatment if possible. It would better reflect what you can see in the WB of some of the cell lines having detectable expression at all. By showing delta delta Ct, this is invisible and it is impossible to see whether cell lines have dramatically different levels of expression.

We have also now included ΔC_T method followed by $2^{-\Delta CT}$ (to understand relative levels of ZFP36L1 compared to *ACTB*) in Supplementary Figs. 17a-c.

8. In the AML cell models at the end of the study, I assume they do not work through the ASCL1 mechanism since it is probably not expressed. Is there some other related factor in the AMLs that could serve this role such as ASCL2?

The reviewer brings up a good point that the mechanism by which ZFP36L1 regulates AML downstream of LSD1 inhibition is distinct as AMLs don't express genes associated with the SCLC neuroendocrine program including ASCL1 and INSM1. We would hypothesize that ZFP36L1 regulates mRNA abundance of AML lineage transcription factors that are necessary to maintain AML in an de-differentiated state. A recent publication performed a CRISPR/Cas9 screen to identify genes that are important for keeping AML in a de-differentiated state and identified LSD1 and ZFP36L2 as inhibitors of differentiation in AML. They found that ZFP36L2 degrades ZFP36L1 to maintain the de-differentiated state of AML implicating ZFP36L1 as an inducer of AML differentiation(Wang, Zhou et al. 2021). This directionality is consistent with our data in SCLC showing that ZFP36L1 opposes the SCLC neuroendocrine phenotype (which is the de-differentiated state of SCLC). We believe that identifying ZFP36L1 targets in AML is very interesting, but not within the scope of this study.

References

Augert, A., E. Eastwood, A. H. Ibrahim, N. Wu, E. Grunblatt, R. Basom, D. Liggitt, K. D. Eaton, R. Martins, J. T. Poirier, C. M. Rudin, F. Milletti, W. Y. Cheng, F. Mack and D. MacPherson (2019). "Targeting NOTCH activation in small cell lung cancer through LSD1 inhibition." Sci Signal **12**(567).

Cai, L., H. Liu, F. Huang, J. Fujimoto, L. Girard, J. Chen, Y. Li, Y. A. Zhang, D. Deb, V. Stastny, K. Pozo, C. S. Kuo, G. Jia, C. Yang, W. Zou, A. Alomar, K. Huffman, M. Papari-Zareei, L. Yang, B. Drapkin, E. A. Akbay, D. S. Shames, Wistuba, II, T. Wang, J. E. Johnson, G. Xiao, R. J. DeBerardinis, J. D. Minna, Y. Xie and A. F. Gazdar (2021). "Cell-autonomous immune gene expression is repressed in pulmonary neuroendocrine cells and small cell lung cancer." Commun Biol **4**(1): 314.

Gay, C. M., C. A. Stewart, E. M. Park, L. Diao, S. M. Groves, S. Heeke, B. Y. Nabet, J. Fujimoto, L. M. Solis, W. Lu, Y. Xi, R. J. Cardnell, Q. Wang, G. Fabbri, K. R. Cargill, N. I. Vokes, K. Ramkumar, B. Zhang, C. M. Della Corte, P. Robson, S. G. Swisher, J. A. Roth, B. S. Glisson, D. S. Shames, Wistuba, II, J. Wang, V. Quaranta, J. Minna, J. V. Heymach and L. A. Byers (2021). "Patterns of transcription factor programs and immune pathway activation define four major subtypes of SCLC with distinct therapeutic vulnerabilities." Cancer Cell **39**(3): 346-360 e347.

Hong, D., E. H. Knelson, Y. Li, Y. T. Durmaz, W. Gao, E. Walton, A. Vajdi, T. Thai, M. Sticco-Ivins, A. H. Sabet, K. L. Jones, A. C. Schinzel, R. T. Bronson, Q. D. Nguyen, M. Y. Tolstorukov, M. Vivero, S. Signoretti, D. A. Barbie and M. G. Oser (2021). "Plasticity in the Absence of NOTCH Uncovers a RUNX2-Dependent Pathway in Small Cell Lung Cancer." Cancer Res.

Huang, Y. H., O. Klingbeil, X. Y. He, X. S. Wu, G. Arun, B. Lu, T. D. D. Somerville, J. P. Milazzo, J. E. Wilkinson, O. E. Demerdash, D. L. Spector, M. Egeblad, J. Shi and C. R. Vakoc (2018). "POU2F3 is a master regulator of a tuft cell-like variant of small cell lung cancer." Genes Dev **32**(13-14): 915-928.

Jin, Y., D. Ma, T. Gramyk, C. Guo, R. Fang, H. Ji and Y. G. Shi (2019). "Kdm1a promotes SCLC progression by transcriptionally silencing the tumor suppressor Rest." Biochem Biophys Res Commun **515**(1): 214-221.

Kanouni, T., C. Severin, R. W. Cho, N. Y. Yuen, J. Xu, L. Shi, C. Lai, J. R. Del Rosario, R. K. Stansfield, L. N. Lawton, D. Hosfield, S. O'Connell, M. M. Kreilein, P. Tavares-Greco, Z. Nie, S. W. Kaldor, J. M. Veal, J. A. Stafford and Y. K. Chen (2020). "Discovery of CC-90011: A Potent and Selective Reversible Inhibitor of Lysine Specific Demethylase 1 (LSD1)." J Med Chem **63**(23): 14522-14529.

Lee, J. K., J. Lee, S. Kim, S. Kim, J. Youk, S. Park, Y. An, B. Keam, D. W. Kim, D. S. Heo, Y. T. Kim, J. S. Kim, S. H. Kim, J. S. Lee, S. H. Lee, K. Park, J. L. Ku, Y. K. Jeon, D. H. Chung, P. J. Park, J. Kim, T. M. Kim and Y. S. Ju (2017). "Clonal History and Genetic Predictors of Transformation Into Small-Cell Carcinomas From Lung Adenocarcinomas." J Clin Oncol **35**(26): 3065-3074.

Lim, J. S., A. Ibaseta, M. M. Fischer, B. Cancilla, G. O'Young, S. Cristea, V. C. Luca, D. Yang, N. S. Jahchan, C. Hamard, M. Antoine, M. Wislez, C. Kong, J. Cain, Y. W. Liu, A. M. Kapoun, K. C. Garcia, T. Hoey, C. L. Murriel and J. Sage (2017). "Intratumoural heterogeneity generated by Notch signalling promotes small-cell lung cancer." Nature **545**(7654): 360-364.

Loh, X. Y., Q. Y. Sun, L. W. Ding, A. Mayakonda, N. Venkatachalam, M. S. Yeo, T. C. Silva, J. F. Xiao, N. B. Doan, J. W. Said, X. B. Ran, S. Q. Zhou, P. Dakle, P. Shyamsunder, A. P. Koh, R. Y.

Huang, B. P. Berman, S. Y. Tan, H. Yang, D. C. Lin and H. P. Koeffler (2020). "RNA-Binding Protein ZFP36L1 Suppresses Hypoxia and Cell-Cycle Signaling." Cancer Res **80**(2): 219-233.
Maes, T., C. Mascaro, I. Tirapu, A. Estiarte, F. Ciceri, S. Lunardi, N. Guibourt, A. Perdones, M. M. P. Lufino, T. C. P. Somervaille, D. H. Wiseman, C. Duy, A. Melnick, C. Willekens, A. Ortega, M. Martinell, N. Valls, G. Kurz, M. Fyfe, J. C. Castro-Palomino and C. Buesa (2018). "ORY-1001, a Potent and Selective Covalent KDM1A Inhibitor, for the Treatment of Acute Leukemia." Cancer Cell **33**(3): 495-511 e412.
Mahadevan, N. R., E. H. Knelson, J. O. Wolff, A. Vajdi, M. Saigi, M. Campisi, D. Hong, T. C. Thai, B. Piel, S. Han, B. B. Reinhold, J. S. Duke-Cohan, M. J. Poitras, L. J. Taus, P. H. Lizotte, A. Portell, V. Quadros, A. D. Santucci, T. Murayama, I. Canadas, S. Kitajima, A. Akitsu, M. Fridrikh, H. Watanabe, B. Reardon, P. C. Gokhale, C. P. Paweletz, M. M. Awad, E. M. Van Allen, A. Lako, X. T. Wang, B. Chen, F. Hong, L. M. Sholl, M. Y. Tolstorukov, K. Pfaff, P. A. Janne, E. Gjini, R. Edwards, S. Rodig, E. L. Reinherz, M. G. Oser and D. A. Barbie (2021). "Intrinsic immunogenicity of small cell lung carcinoma revealed by its cellular plasticity." Cancer Discov.
Oser, M. G., M. J. Niederst, L. V. Sequist and J. A. Engelman (2015). "Transformation from non-small-cell lung cancer to small-cell lung cancer: molecular drivers and cells of origin." Lancet Oncol **16**(4): e165-172.
Oser, M. G., A. H. Sabet, W. Gao, A. A. Chakraborty, A. C. Schinzel, R. B. Jennings, R. Fonseca, D. M. Bonal, M. A. Booker, A. Flaifel, J. S. Novak, C. L. Christensen, H. Zhang, Z. T. Herbert, M. Y. Tolstorukov, E. J. Buss, K. K. Wong, R. T. Bronson, Q. D. Nguyen, S. Signoretti and W. G. Kaelin, Jr. (2019). "The KDM5A/RBP2 histone demethylase represses NOTCH signaling to sustain neuroendocrine differentiation and promote small cell lung cancer tumorigenesis." Genes Dev **33**(23-24): 1718-1738.
Schoenherr, C. J. and D. J. Anderson (1995). "The neuron-restrictive silencer factor (NRSF): a coordinate repressor of multiple neuron-specific genes." Science **267**(5202): 1360-1363.
Sequist, L. V., B. A. Waltman, D. Dias-Santagata, S. Digumarthy, A. B. Turke, P. Fidias, K. Bergethon, A. T. Shaw, S. Gettinger, A. K. Cosper, S. Akhavanfard, R. S. Heist, J. Temel, J. G. Christensen, J. C. Wain, T. J. Lynch, K. Vernovsky, E. J. Mark, M. Lanuti, A. J. Iafrate, M. Mino-Kenudson and J. A. Engelman (2011). "Genotypic and histological evolution of lung cancers acquiring resistance to EGFR inhibitors." Sci Transl Med **3**(75): 75ra26.
Sheng, W., M. W. LaFleur, T. H. Nguyen, S. Chen, A. Chakravarthy, J. R. Conway, Y. Li, H. Chen, H. Yang, P. H. Hsu, E. M. Van Allen, G. J. Freeman, D. D. De Carvalho, H. H. He, A. H. Sharpe and Y. Shi (2018). "LSD1 Ablation Stimulates Anti-tumor Immunity and Enables Checkpoint Blockade." Cell **174**(3): 549-563 e519.
Wang, E., H. Zhou, B. Nadorp, G. Cayanan, X. Chen, A. H. Yeaton, S. Nomikou, M. T. Witkowski, S. Narang, A. Kloetgen, P. Thandapani, N. Ravn-Boess, A. Tsirigos and I. Aifantis (2021). "Surface antigen-guided CRISPR screens identify regulators of myeloid leukemia differentiation." Cell Stem Cell **28**(4): 718-731 e716.

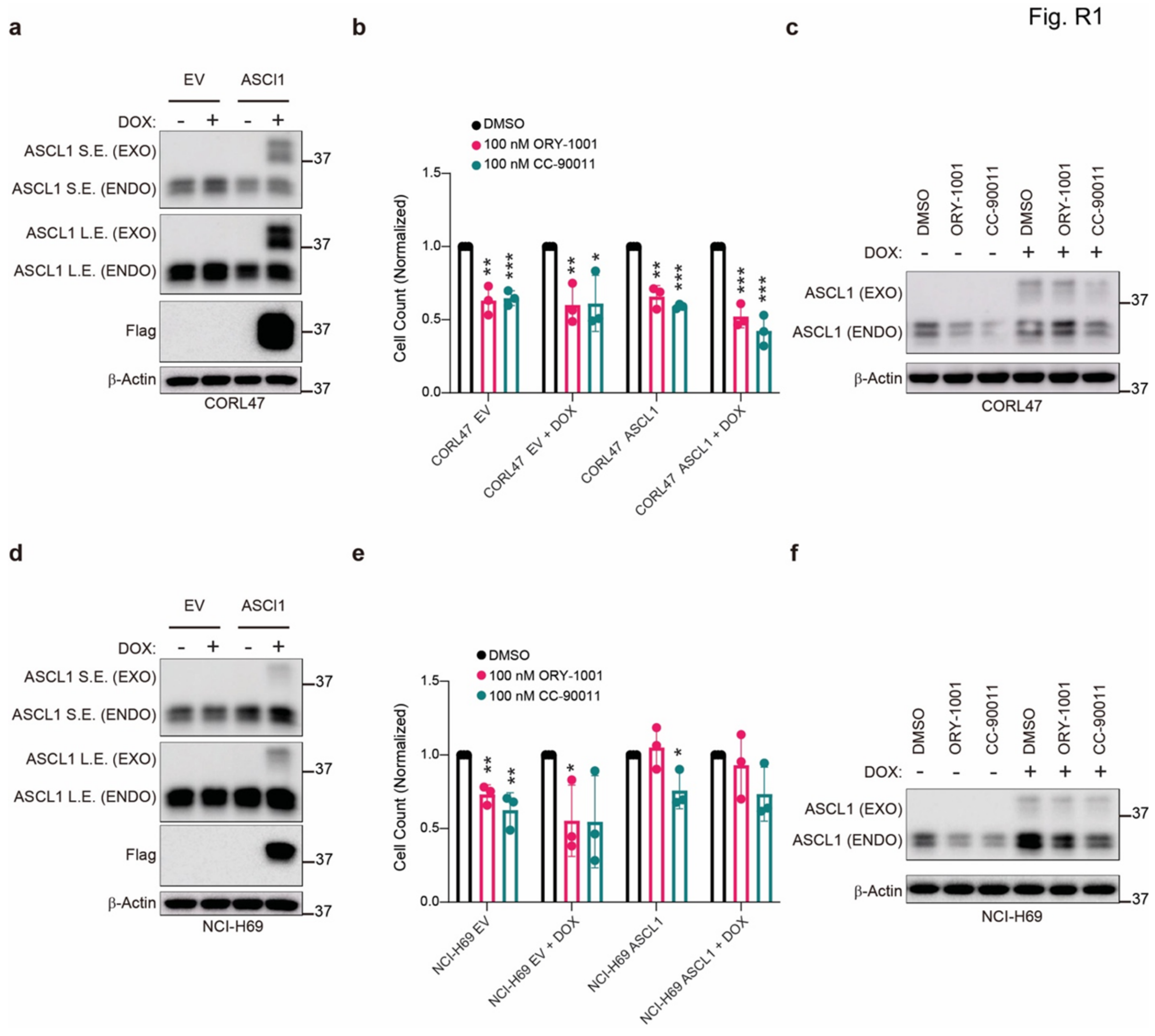

Fig. R1. CORL47 and NCI-H69 Cells Engineered to Inducibly Express Exogenous ASCL1 and Then Treated with the LSD1 inhibitors ORY-1001 or CC-90011

(a and d) Immunoblot analysis of CORL47 cells (**a**) or NCI-H69 cells (**d**) stably infected with a doxycycline inducible (DOX-ON) lentivirus encoding ASCL1-FLAG or the corresponding empty vector (EV) and then treated with DOX (2 μg/mL) for 3 days where indicated. (**b and e**) Cell counts of ASCL1 isogenic CORL47 cells (**b**) or NCI-H69 cells (**e**) from a treated with or without DOX as above where indicated and treated with the LSD1 inhibitors ORY-1001 or CC-

90011 at 100 nM for 12 days. n=3 biological replicates. (**c and f**) Immunoblot analysis of CORL47 (**c**) or NCI-H69 (**f**) DOX-ON ASCL1-FLAG cells after treatment with ORY-1001 or CC-90011 for 12 days. EXO indicates exogenous ASCL1. ENDO indicates endogenous ASCL1. Note exogenous ASCL1 expression is very weak compared to endogenous ASCL1 expression.

Fig. R2

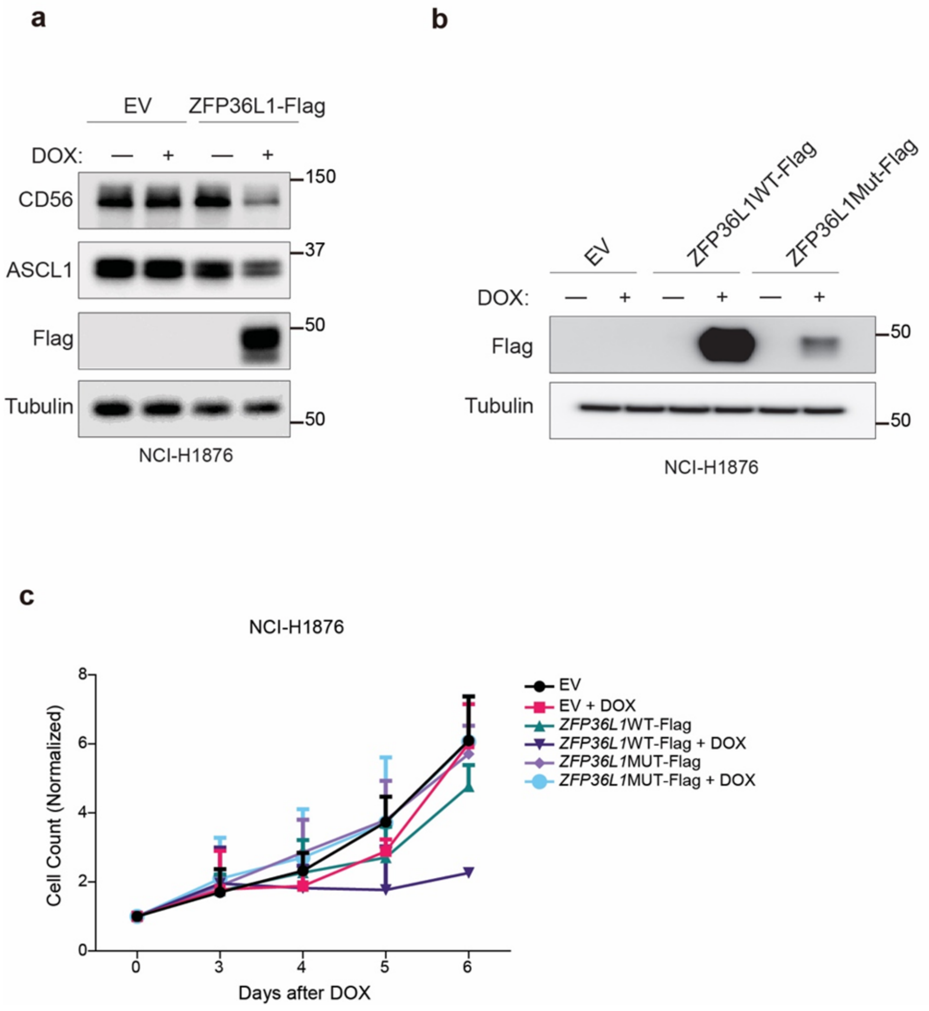

Fig. R2. NCI-H1876 Cells Engineered to Inducibly Express Exogenous ZFP36L1 WT or an mRNA-Binding Mutant

(a) Immunoblot analysis of NCI-H1876 cells infected with a doxycycline inducible (DOX-ON) lentivirus encoding ZFP36L1WT-FLAG or the corresponding empty vector (EV) and then treated with DOX (1 μg/mL) for 3 days where indicated. Note that ASCL1 and CD56 (which is NCAM1, another neuroendocrine marker) goes down when ZFP36L1 WT is expressed. (**b**) Immunoblot analysis of NCI-H1876 cells infected with a doxycycline inducible (DOX-ON) lentivirus encoding ZFP36L1WT-FLAG, the ZFP36L1 mRNA-binding mutant, or the corresponding empty vector (EV) and then treated with DOX (1 μg/mL) for 3 days where

indicated. Note that expression of the mRNA-binding mutant is markedly weaker than the WT, which confounds the interpretation of the results in Fig. c. (**c**) Cell proliferation assays of the NCI-H1876 ZFP36L1 isogenic cell lines in b treated with DOX (1 μg/mL) for the days indicated. Note that although ZFP36L1-WT does suppress proliferation of the cells, it is difficult to interpret whether the mutant rescues the phenotype given it’s expressed at a markedly lower level.

Fig. R3

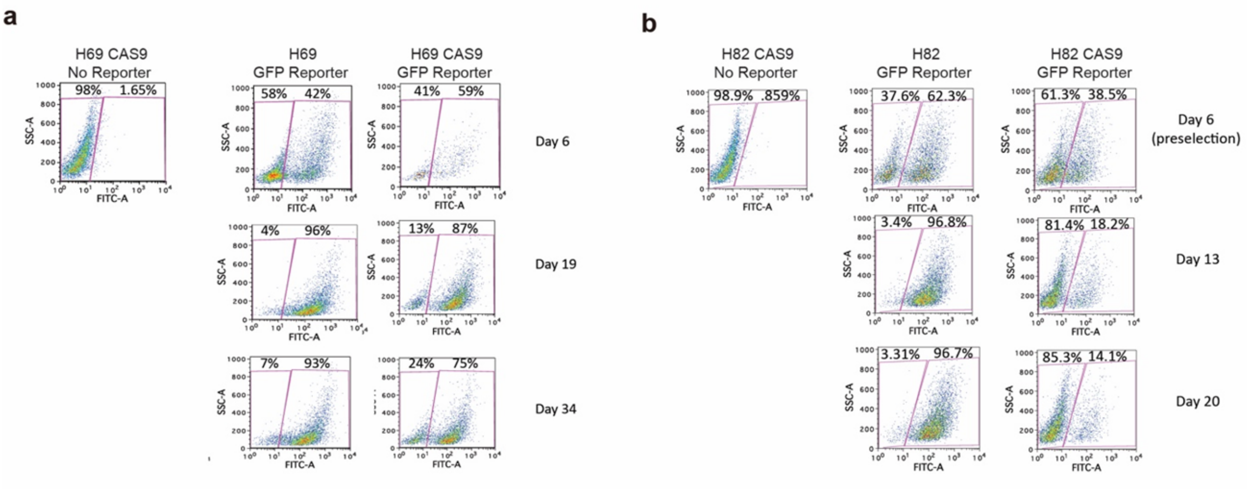

Fig. R3. Cas9 Activity Reporter Assays in NCI-H69 and NCI-H82 Cells

(**a and b**) NCI-H69 (**a**) or NCI-H82 (**b**) parental cells were engineered to stably express Cas9 or not and then superinfected with a GFP reporter lentivirus that quantifies Cas9 activity. The GFP reporter lentivirus contains GFP and an sgRNA against GFP and therefore Cas9 editing will silence GFP if it is effective. Note that Cas9 activity in NCI-H69 cells shows extremely poor editing and only edits ~17% of total cells over a 34 day period. NCI-H82 cells are included as a control for high Cas9 efficiency editing and edits ~82% of cells over 20 days. The inherent low Cas9 activity in NCI-H69 cells precludes NCI-H69 cells from being able to be used to generate knockouts in CRISPR/Cas9 experiments.

REVIEWERS' COMMENTS

Reviewer #1 (Remarks to the Author):

After reviewing the revised manuscript and authors response to all reviewers, the manuscript is recommended for publication.

Reviewer #2 (Remarks to the Author):

The authors have adequately addressed my concerns in the revised manuscript. I support publication at Nature Communications.

Reviewer #3 (Remarks to the Author):

The authors have addressed my comments and the other reviewer comments thoroughly, strengthening an already strong study.
